# Characterization of a pathway of genomic instability induced by R-loops and its regulation by topoisomerases in *E*. *coli*

**DOI:** 10.1371/journal.pgen.1010754

**Published:** 2023-05-04

**Authors:** Julien Brochu, Émilie Vlachos-Breton, Dina Irsenco, Marc Drolet

**Affiliations:** Département de microbiologie, infectiologie et immunologie, Université de Montréal, Montréal, Canada; Duke University, UNITED STATES

## Abstract

The prototype enzymes of the ubiquitous type IA topoisomerases (topos) family are *Escherichia coli* topo I (*topA*) and topo III (*topB*). Topo I shows preference for relaxation of negative supercoiling and topo III for decatenation. However, as they could act as backups for each other or even share functions, strains lacking both enzymes must be used to reveal the roles of type IA enzymes in genome maintenance. Recently, marker frequency analysis (MFA) of genomic DNA from *topA topB* null mutants revealed a major RNase HI-sensitive DNA peak bordered by *Ter*/Tus barriers, sites of replication fork fusion and termination in the chromosome terminus region (Ter). Here, flow cytometry for R-loop-dependent replication (RLDR), MFA, R-loop detection with S9.6 antibodies, and microscopy were used to further characterize the mechanism and consequences of over-replication in Ter. It is shown that the Ter peak is not due to the presence of a strong origin for RLDR in Ter region; instead RLDR, which is partly inhibited by the backtracking-resistant rpoB*35 mutation, appears to contribute indirectly to Ter over-replication. The data suggest that RLDR from multiple sites on the chromosome increases the number of replication forks trapped at *Ter*/Tus barriers which leads to RecA-dependent DNA amplification in *Ter* and to a chromosome segregation defect. Overproducing topo IV, the main cellular decatenase, does not inhibit RLDR or Ter over-replication but corrects the chromosome segregation defect. Furthermore, our data suggest that the inhibition of RLDR by topo I does not require its C-terminal-mediated interaction with RNA polymerase. Overall, our data reveal a pathway of genomic instability triggered by R-loops and its regulation by various topos activities at different steps.

## Author summary

DNA topoisomerases act during replication, transcription, and recombination to solve topological problems inherent to the double-helical structure of DNA. Topos of the type 1A family are the only ones that are ubiquitous. The prototype enzymes of the two main type IA subfamilies are topo I and topo III from *Escherichia coli*. The recent finding that *E*. *coli* topo I and III can suppress R-loop formation suggested that R-loops have been a problem early in the evolution of life. However, how toxic R-loops are generated and how they exert their detrimental effect on genome stability, especially in the absence of type IA topos, is still largely unknown. Here, we have uncovered a pathway leading to genome instability in the absence of type IA topos: unregulated replication from R-loops involving RNAP backtracking leads to DNA amplification in the chromosome terminus region due to replication forks blocked at termination barriers. Furthermore, our data suggest that type IA topos play major roles at the termination step of replication when convergent replication forks merge and R-loops exert their detrimental effects mostly via topological problems. Overall, our data shed new light on how R-loops can lead to genomic instability and how topos can deal with this problem.

## Introduction

Because of the double-helical structure of DNA, each time the two strands are separated during replication, transcription, or repair, underwinding and overwinding (supercoiling) occur. Such excess supercoiling, in turn, interferes with normal gene expression and replication. Furthermore, intertwining of the DNA occurs during replication and repair. If not correctly resolved, such intertwining inhibit chromosome segregation and may lead to DNA breaks and genomic instability. To solve these topological problems, cells possess DNA topoisomerases (topos), which are nicking-closing enzymes [1,2], which cut one (type I) or two (type II) DNA strands. Type I and II are further divided respectively into types IA and IB and IIA and IIB. To solve topological problems, type IA and type II enzymes use a strand-passage mechanism whereas enzymes of the type IB family, also named swivelases, use a rotation mechanism.

Topos of the type IA family are the only ones that are ubiquitous [3,4]. They use a strand-passage mechanism to relax or decatenate DNA. Unlike other topos, they use ssDNA as substrates and many of them possess RNA topo activity [3,5,6]. Type IA topos are classified into three subfamilies, the two main ones being topo I and topo III with *E*. *coli topA* and *topB* encoding the prototype enzyme of these two subfamilies. Topo I is present in all bacteria but not in archaea and eukaryotes, whereas topo III is found in most bacteria and in all archaea and eukaryotes. Topo III has a higher requirement for ssDNA than topo I and mostly acts as a decatenase [7], whereas topo I mainly acts to relax negative supercoiling [4]. The results of recent single-molecule experiments suggest that a dynamic fast gate for topo I may promote efficient relaxation of negatively supercoiled DNA. In contrast, a slower gate-closing for topo III may facilitate capture of dsDNA and, as a result, efficient decatenation [8].

Null mutations in the *topA* gene of *E*. *coli* inhibit cell growth. Compensatory mutations may arise that allow *topA* null cells to generate visible colonies (reviewed in [4]). Such mutations are either substitutions in *gyrA* or *gyrB* genes that reduce the negative supercoiling activity of gyrase [9,10], or amplification of a chromosomal region allowing topo IV overproduction [4,11–14]. DNA gyrase introduces negative supercoiling, via the relaxation of positive supercoiling generated during replication [15] and transcription [16]. The main function of topo IV is to act as a decatenase behind the replication fork to remove precatenanes and, once chromosomal replication is completed, to remove catenanes to allow chromosome segregation [17,18]. Evidence of a minor role of topo IV in the regulation of chromosome topology via the relaxation of negative supercoiling has been reported [19]. Topo I interacts with RNA polymerase (RNAP) [20] and relaxes negative supercoiling generated behind moving RNAPs during transcription [21]. The failure to relax transcription-induced negative supercoiling in *topA* null mutants leads to hypernegative supercoiling and the formation of R-loops that can inhibit gene expression and activates R-loop-dependent replication (RLDR hereafter) [22–29]. Thus, one major function of topo I in *E*. *coli* is the relaxation of transcription-induced negative supercoiling to inhibit R-loop formation. Genome-wide approaches have recently been used to demonstrate this major function of topo I [30].

Unlike *topA* null mutants, *topB* null cells grow as well as wild-type cells, but show a delayed and disorganized nucleoid segregation phenotype, as compared with wild-type cells [31]. Topo III is found to be associated with replication forks *in vivo* (via an interaction with SSB and DnaX; [31,32]), where its substrate, ssDNA, is present. *In vitro*, topo III has a strong decatenation activity that can fully support replication including the termination step [33]. Thus, it is very likely that topo III plays a role in the removal of pre-catenanes during replication [31,34]. However, at least when the activity of other topoisomerases is not disturbed, this role appears to be relatively minor compared with topo IV, the absence of which inhibits growth and causes severe chromosome segregation defects (the *par* phenotype [13]). In fact, when topo IV [35,36] or gyrase [37] activity is significantly perturbed, topo III becomes essential for decatenation.

**Fig 1:**
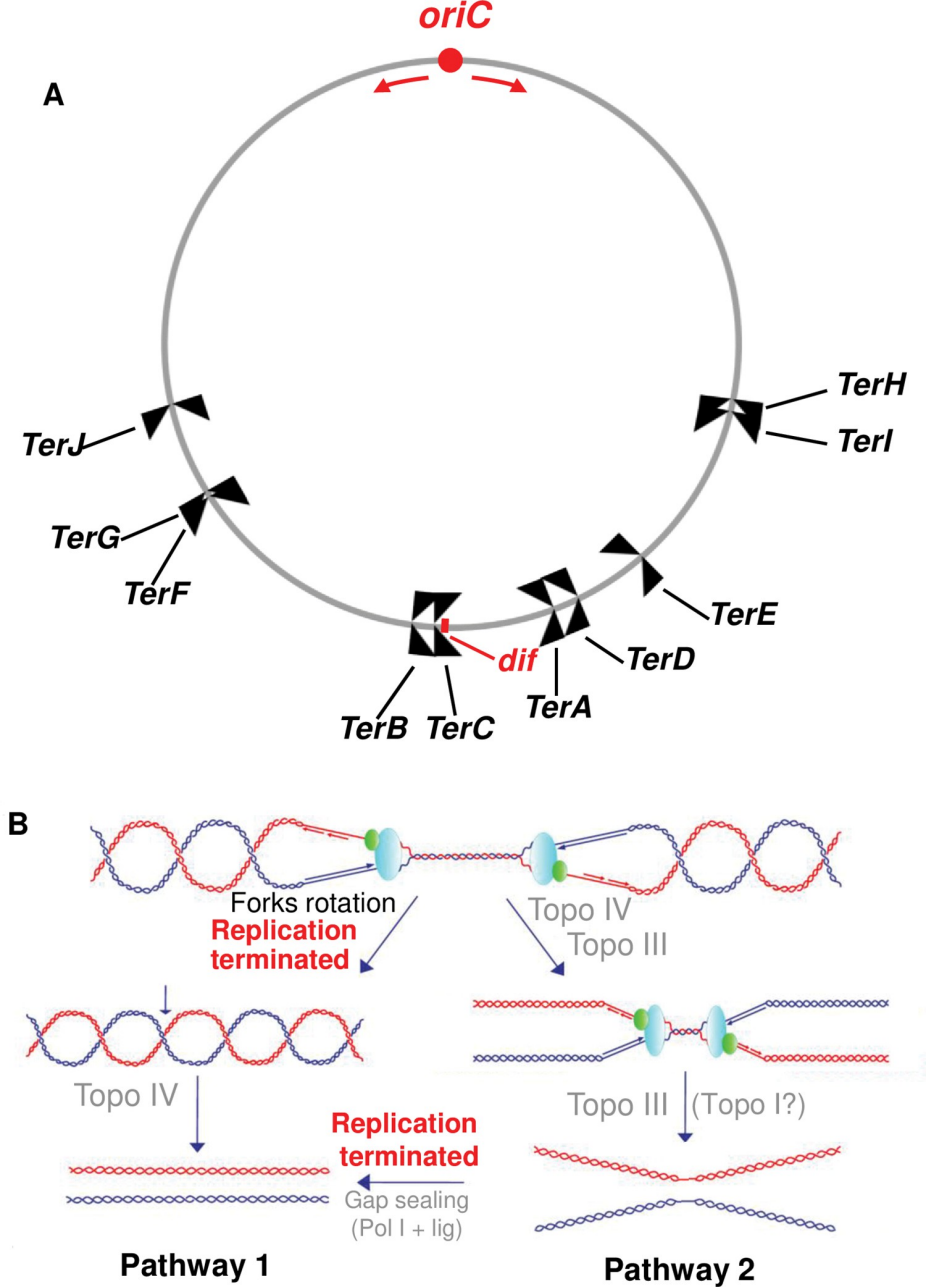
The chromosome terminus (Ter) region of *Escherichia coli*. (A) Representation of the circular chromosome of *E*. *coli* with the *oriC* region (red dot) from which bidirectional replication is initiated (red arrows). The convergent replication forks normally meet in the region opposite to *oriC* (*dif*). *TerA* to *TerJ* are polar *Ter*/Tus barriers that block forks coming from only one direction (indicated by the black arrows). See text for more details. (B) Topological problems associated with replication termination when two replication forks converge. Topo III and topo IV can remove pre-catenanes. In pathway 1 (the main one), replication is fully terminated before the full unlinking of the replicated sister chromosomes by topo IV. In pathway 2, the sister chromosomes are fully unlinked by a type IA topo (mostly topo III) before the completion of the replication. Topo III is shown in green acting at the fork. See text for details.

Bidirectional replication of the circular *E*. *coli* chromosome is initiated at *oriC* and terminated within the opposite broad terminus (Ter) region, where the two convergent replication forks meet and fuse. The final chromosome decatenation step by topo IV and the resolution of chromosome dimers by XerCD take place within this region at the *dif* site [38]. The trapping of replication forks by Tus proteins that binds to polar *Ter* sequences (10 of them, *TerA* to *TerJ*, Fig 1A) restrains the termination process to Ter [38]. *Ter* sequences within the left portion of the chromosome block clockwise-moving forks, whereas those in the right portion of the chromosome block counterclockwise-moving forks. Under normal growth conditions, replication forks merge at the position opposite to *oriC* [39] or very close to this position, where the clockwise fork is first arrested at *TerC* [38]. The process of replication termination is more complex than initially believed in part because fork fusion can lead to over-replication as originally shown *in vitro* [40]. In fact, several proteins including RecG, RecBCD, ligase A, Pol I, RNase HI, Exo I, Exo VII, SbcCD, and RecJ nucleases appear to be involved in the processing of replication termination intermediates [41–50]. The inactivation of many of these proteins alone or in combination has been shown to lead to over-replication to various extents in the Ter region. This hallmark phenotype is characterized by the appearance of a prominent Ter peak bordered by the innermost *Ter*/Tus barrier as shown by MFA by next-generation sequencing (NGS) [41,42,44–46,48,50,51]. Termination involving a *Ter*/Tus barrier normally prevents over-replication [40]. However, when the arrested fork is stuck at the barrier before the arrival of the convergent fork it can lead to over-replication [44,52]. *Ter*/Tus-dependent and independent Ter over-replication requires RecA for D-loop formation that is used for PriA-dependent primosome assembly [41,43,51].

Experimental evidence has been presented which suggests that type IA topos might be involved in the process of replication termination. In one report, it was shown that excess negative supercoiling caused by a mutation in the C-terminal domain of topo I could reduce the ability of Tus to arrest replication forks [53]. The process of replication termination when two replication forks converge represents a major topological challenge (Fig 1B). Indeed, when the converging forks are about to merge, not only does positive supercoiling build-up to very high levels but the space on the DNA template also becomes too small to accommodate the binding of DNA gyrase molecules. At this stage, two alternative pathways [4,32] can be used to complete replication while allowing the last intertwines to be removed to permit chromosome segregation. In the first major pathway, replication forks rotation allows the positive supercoiling to migrate behind the forks as pre-catenanes that are converted to catenanes once replication is completed. These catenanes are removed by topo IV (Pathway 1). In the second pathway, the parental strands are unlinked by topo III before the completion of replication e.g., by pol I to fill the gaps [32,38]. Whether this pathway is used in *E*. *coli* is currently unknown.

Despite much being known about the biochemical properties and main functions of type IA topos in *E*. *coli*, much less is known about the consequences of losing their activity on genome stability. Elucidating the roles of type IA topos in genome maintenance is important, as it may reveal new pathways for genomic instability and help explain the conservation of these enzymes through evolution. Moreover, inhibitors of type IA topos are being developed to be used as antibiotics [54]. The finding that deleting both *topA* and *topB* in *E*. *coli* generated a new phenotype, suggested that either both enzymes share a common function, can act as backups for each other and/or a combination of defects attributed to the absence of topo I or topo III was responsible for this new phenotype: extensive cellular filamentation with a *recA*-dependent chromosome segregation defect [55]. This phenotype was recently correlated with RecA- and R-loop-dependent extensive over-replication in Ter region [14]. Here, it is shown that the involvement of RLDR is largely indirect and likely related to the accumulation of replication forks reaching *Ter*/Tus barriers that in turn increases the likelihood of over-replication in *Ter*. Furthermore, the data suggest that the lack of type IA topos decatenation activity during replication elongation and forks fusion (pathway 2) stimulate over-replication. This causes a high amount of DNA accumulation within the small *TerA*-*TerB* interval including *dif* that leads to a chromosome segregation defect corrected by topo IV overproduction. Overall, our data suggest that the lack of type IA topos may simulate Ter over-replication via excess negative supercoiling (mostly *topA*) that promotes RLDR and defective processing of forks at *Ter*/Tus barriers, and via the lack of decatenation activity (mostly *topB*) during replication elongation and termination.

## Results

### A deletion approach failed to reveal strong RLDR origins that could be responsible for Ter over-replication in *topA topB* null cells

RLDR was first observed in *rnhA* null cells as a kind of replication that was resistant to protein synthesis inhibition (constitutive stable DNA replication (cSDR), as opposed to replication from *oriC* [56,57]. MFA experiments with 150 to 200 kb long hybridization probes initially revealed a peak in Ter region and led the authors to conclude for the presence of origins for RLDR (*oriKs*) in this region [58]. More recently, in a series of experiments with MFA by NGS, the authors were able to precisely delineate the Ter peak in *rnhA* null mutants, but no evidence was found for the presence of well-defined RLDR origins in this region [50]. In fact, in a laboratory evolution experiment, the only strong RLDR origin that could support good growth of a *rnhA* null mutant without the *oriC*/DNA system, was localized far away from the Ter region [59]. These recent results, together with the finding that Ter over-replication is often caused by defective replication termination, led us to investigate in more details the source of this over-replication in *topA topB* null mutants.

**Fig 2:**
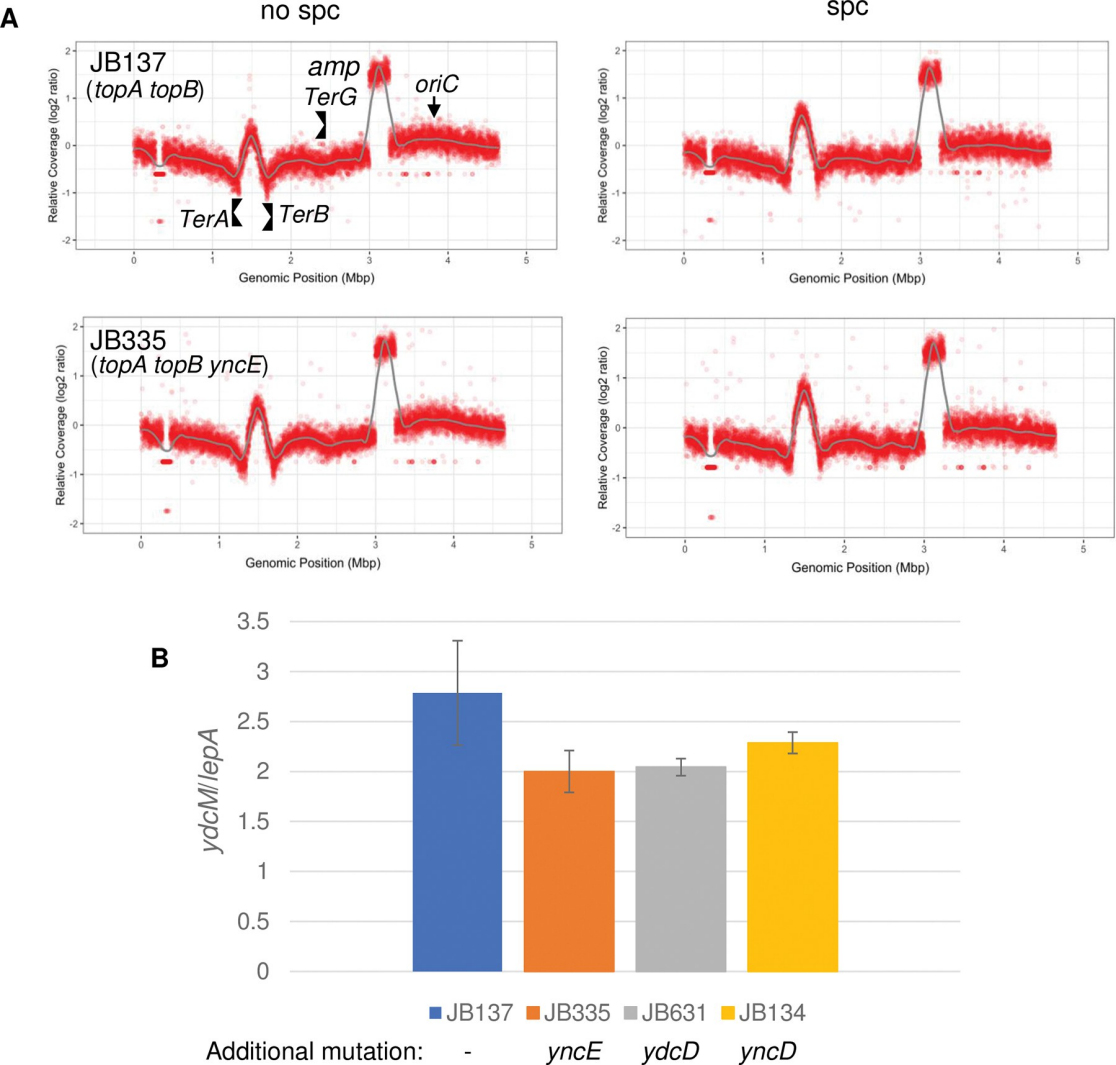
MFA and qPCR show that deleting genes located at the top of the Ter peak in *topA topB* null cells had no effects on the peak height. (A) MFA by NGS of genomic DNA extracted from JB137 (*ΔtopB topA20*::Tn*10 gyrB*(Ts)) and JB335 (*ΔtopB ΔyncE topA20*::Tn*10 gyrB*(Ts)) cells grown at 30°C to log phase and treated (spc) or not treated (no spc) with spectinomycin for two hours. The absolute read counts (Log2) were plotted against chromosomal coordinates (W3110 genomic sequence AP009048.1). The gray line is the loess regression curve. *amp* indicates the amplified *parC parE* region. The *oriC* region as well as *TerA*, *TerB* and *TerG* barriers, are shown. The gap at position around 0.3 corresponds to the Δ(*codB-lacI)3* deletion in these strains. (B) *ydcM/lepA* ratio determined by qPCR of genomic DNA extracted from JB137 (*ΔtopB topA20*::Tn*10 gyrB*(Ts)), JB335 (*ΔtopB ΔyncE topA20*::Tn*10 gyrB*(Ts)), JB631 (*ΔtopB ΔydcD topA20*::Tn*10 gyrB*(Ts)) and JB134 (*ΔtopB ΔyncD topA20*::Tn*10 gyrB*(Ts)) cells grown at 30°C to log phase as described in Material and Methods. Additional mutation, refers to the additional mutation under study that is also present in the *topA topB gyrB*(Ts) strain.

If present, a strong origin (*oriK*) of bi-directional replication should be located at the top of the Ter peak. The highest copy number identified from the MFA data by NGS for *topA topB* null cells is in a region close to the genomic position 1.52 [14], where the two divergently transcribed genes *yncD* and *yncE* are found. *topA topB* null strains carrying either a *yncD* (JB134) or *yncE* (JB335) deletion were first constructed. The *topA* and *topA topB* strains that were used previously and in the present study unless otherwise indicated, carry a *gyrB*(Ts) allele [14,27–29]. Such strains grow better at 37°C than at 30°C owing to the partial inactivation of GyrB, reducing the negative supercoiling activity of gyrase. Temperature downshifts to 30°C rapidly re-activates gyrase activity which lead to the full expression of *topA* null phenotypes, including slow growth, hypernegative supercoiling and high level of R-loop formation and RLDR [14,22,27,29]. In our studies, these strains were grown at 30°C (see Material and Methods). Fig 2A shows the MFA profiles by NGS of genomic DNA from JB137 (*topA topB*) and JB335 (*topA topB yncE*) strains. Spc means that spectinomycin was added to log phase cells for two hours before genomic DNA extraction as previously done [14]. After two hours, *oriC*-dependent replication is fully terminated, whereas RLDR is still active. The Ter peak heights, regardless of whether *spc* was added, were found to be indistinguishable between the strains, indicating that *yncE* plays no role in Ter over-replication (Fig 2A). As expected, since RLDR was still active, the Ter peak height increased after the spc treatment as previously observed [14]. The *ydcM/lepA* ratio by qPCR with genomic DNA from JB137 (*topA topB*), JB134 (*topA topB yncD*) and JB335 (*topA topB yncE*) was also determined. *ydcM* is located close to the top of the Ter peak and *lepA* is outside Ter, and it was previously shown that the *ydcM/lepA* ratio accurately reflected the Ter peak height revealed by MFA [14]. Fig 2B shows that the *ydcm/lepA* ratios were very similar between the strains [14]. Thus, *yncE* and *yncD* plays no significant roles in Ter over-replication.

Preliminary DRIP-seq data to map R-loops genome-wide in *topA topB* null mutants revealed a good candidate for a possible *oriK*, close to the top of the Ter peak. R-loop formation in *topA topB* null cells at this site, within *ydcD* (close to 1.53), was confirmed by DRIP-qPCR as shown in S1 Fig. The *topA topB ydcD* strain (JB631) was constructed and the *ydcM/lepA* ratio was determined and found to be very similar to that of strains JB137, JB134 and JB335 (Fig 2B). Thus, despite R-loop formation at this site, deleting it had no effect on the Ter peak height. Together, these results did not reveal the presence of strong *oriK* sites at the top of the Ter peak that could be responsible for over-replication in this region.

### Deleting *tus* generated a flattened profile in MFA with no Ter peaks, improved growth and significantly corrected the chromosome segregation defect of a *topA topB* null mutant

**Fig 3:**
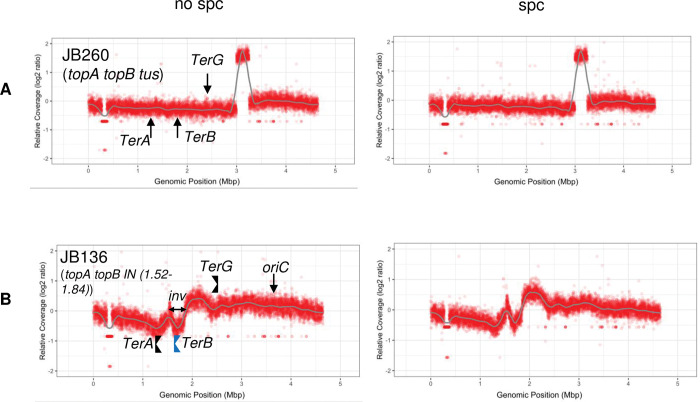
MFA shows that deleting *tus* and inverting the *TerB*/Tus barrier, respectively, eliminates and re-localizes the high peak in *topA topB* null cells. MFA by NGS of genomic DNA extracted from (A) JB260 (*ΔtopB ΔtusB topA20*::Tn*10 gyrB*(Ts)) and (B) JB136 (*ΔtopB ΔyncE topA20*::Tn*10 gyrB*(Ts) *IN(1*.*52–1*.*84)*) cells grown at 30°C to log phase and treated (spc) or not treated (no spc) with spectinomycin for two hours. The inverted *TerB* barrier in JB136 is shown in blue (*TerB* is located within the *IN(1*.*52–1*.*84)* inversion in JB136 (*inv*)). See legend to Fig 2 for more details.

Another approach was used to investigate for the presence of strong *oriKs* in the Ter region. MFA profiles show that counterclockwise and clockwise moving replication forks within the Ter region are blocked by the *TerA*/Tus and *TerB*/Tus barriers, respectively (e.g. JB137, Fig 2A). Thus, if strong *oriKs* are present in the Ter region, deleting *tus* should lead to a clear reduction in Ter peak height but the peak should still be visible, whereas if no active *oriKs* are present a flattened profile should be generated. Fig 3A shows that the MFA profile of a *topA topB tus* strain (JB260, no spc) is clearly flattened with no peak at the genomic position identified in strain JB137 (Fig 2A). The absence of significantly active *oriKs* in the Ter region is further confirmed by the profile of spectinomycin-treated cells (JB260, spc), where replication from *oriC* was fully inhibited and RLDR was still active (Fig 3A). That RLDR was active in JB260 cells is shown below. A similar result, a flattened MFA profile in the Ter region upon *tus* deletion, was the strongest evidence for the absence of *oriKs* in the Ter region of *rnhA* null cells [50]. The JB260 MFA profiles (Fig 3A) also show the absence of significantly active *oriKs* outside the Ter region. Thus, these results show that: 1- RLDR could be the result of weak and widely distributed *oriKs* across the genome that are stochastically activated in the cell population as previously suggested [60] and 2- The presence of the prominent Ter peak in *topA topB* null cells could reflect over-replication associated with the process of replication fork fusion at *Ter*/Tus barriers. This is supported by the data in the next section. In this context, RLDR may contribute to over-replication by increasing the number of forks trapped at *Ter*/Tus barriers for a long period of time.

**Fig 4:**
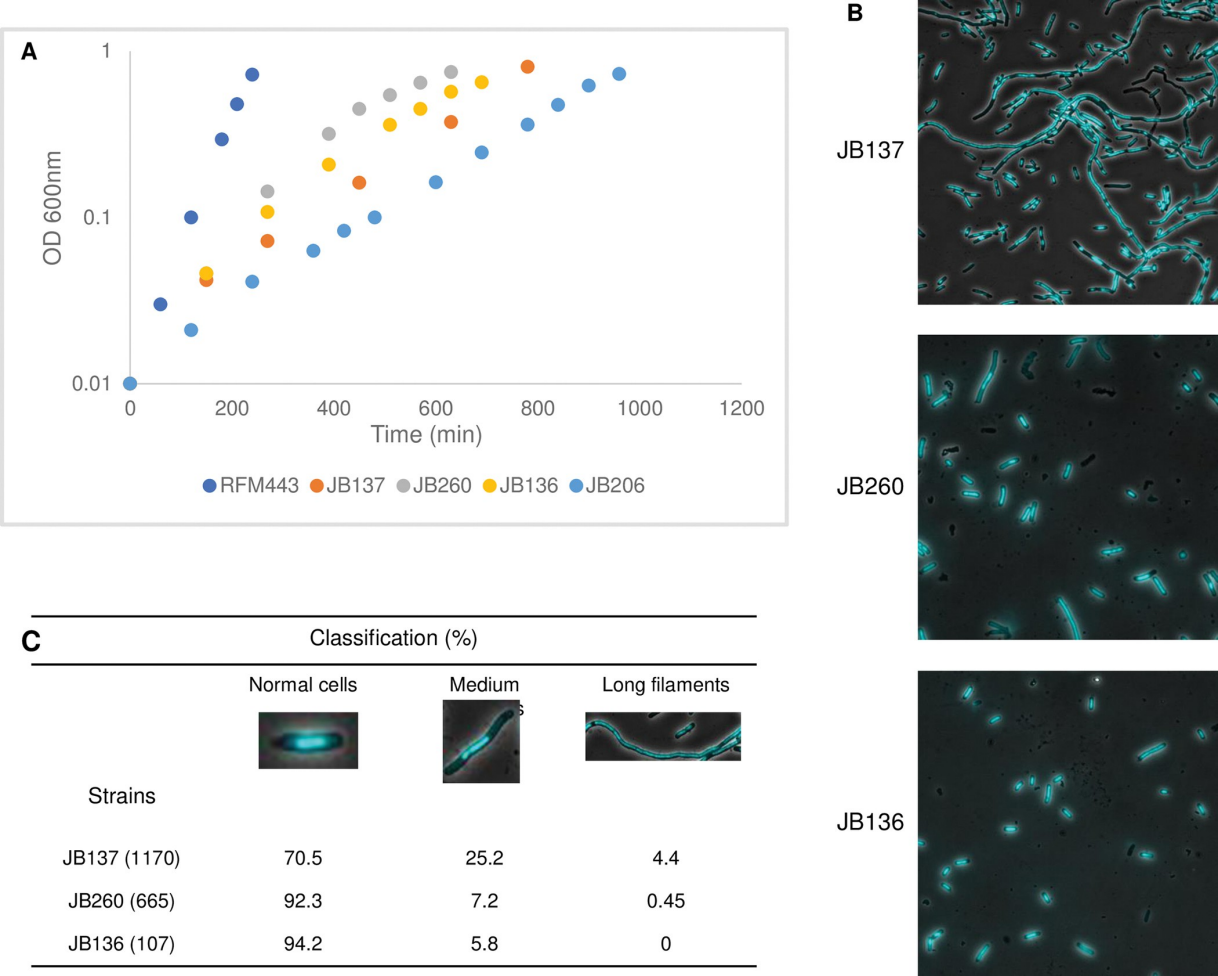
Deleting *tus* or inverting the *TerB* barrier slightly improve growth and significantly correct the filamentation and chromosome segregation defects of *topA topB* null cells. Cells of RFM443 (wild-type), JB137 (*ΔtopB topA20*::Tn*10 gyrB*(Ts)), JB260 (*ΔtopB ΔtusB topA20*::Tn*10 gyrB*(Ts)), JB136 (*ΔtopB ΔyncE topA20*::Tn*10 gyrB*(Ts) *IN(1*.*52–1*.*84)*), and JB206 (*topA20*::Tn*10 gyrB*(Ts)) strains were grown overnight at 37°C on LB plates and diluted in fresh liquid LB medium for growth curve at 30°C (A) or for growth at 30°C to an OD_600_ of 0.8 for microscopy (B), as described in Material and Methods. Representative merged images of phase contrast and fluorescence pictures of SYTO-40-stained cells are shown in (B). In (C), cells for each strain (total number in parentheses) were examined in merged images to calculate the percentage of cells in each category.

Growth, cellular morphology, and nucleoid shape parameters of JB260 (*topA topB tus*) were compared with those of JB137 (*topA topB*). Fig 4A shows that JB260 cells grew significantly better than JB137 cells. Microscopy analysis shows that JB137 cells were generally much longer than JB260 cells and that their DNA was not evenly distributed (Fig 4B for pictures and 4C for quantitative analysis). Thus, these results strongly suggest that the growth and chromosome segregation defects of *topA topB* null cells are largely related to over-replication in the Ter region.

### Analysis of the MFA profile of a *topA topB* null strain carrying a DNA inversion including *TerB* supports the hypothesis of over-replication being triggered by fork fusion events at *Ter*/Tus barriers

While we were constructing *topA topB yncE* strains, we isolated a clone that had a much lower *ydcM/lepA* ratio than other *topA topB yncE* clones. This clone, strain JB136, carries an inversion that changes the position of the over-replicated region normally located in Ter (see Materials and Methods). The MFA profile revealed that the inverted region in JB136 includes *TerB*, so that this site now becomes the left boundary of the over-replicated region whereas *TerG*, a weaker barrier [38], is now the right boundary (Fig 3B, JB136). The high level of DNA replication on the right side of *TerG* illustrates both the weak activity of this site as a barrier and the high level of replication originating from the *TerB-TerG* interval. Strikingly, the peak between *TerB* and *TerG* in JB136 was as high as that between *TerA* and *TerB* in JB137 (compare JB137, Fig 2A, with JB136, Fig 3B), despite the lack of *oriKs* in this area as shown in the absence of a *Ter*/Tus barriers (Fig 3A, JB260, *topA topB tus*, no prominent peaks between *TerB* and *TerG*; no significant peaks also in this area in JB137 (Fig 2A)). This mimics the situation with JB137, where the absence of *Tus* (JB260) eliminated the prominent peak between *TerA* and *TerB*. It can also be seen that the copy number is still very high after *TerG* for more than 1 Mbp toward *oriC* and that in fact the highest copy number is not located at *oriC* (Fig 3B, spc). This is consistent with the presence of replication forks moving toward this *oriC* region. This is better illustrated by the MFA profile of JB136 relative to JB137 (S2 Fig). Altogether, our results are consistent with a model in which over-replication is mostly triggered by replication forks trapped at *Ter*/Tus barriers (mainly *TerB* here).

Growth, cellular morphology, and nucleoid shape parameters of JB136 (*topA topB ynce IN(1*.*52–1*.*84)*) were compared with those of JB137 (*topA topB*). JB136 grew slightly better than JB137 (Fig 4A) whereas JB137 cells were generally much longer than JB136 cells and their DNA was not evenly distributed (Fig 4B for pictures and 4C for quantitative analysis). These results show that the chromosome segregation defect of *topA topB* null cells is mostly due to over-replication specifically in the Ter region.

### The absence of topo III in *topA* null cells dramatically increase the Ter peak height but not R-loop formation and RLDR

**Fig 5:**
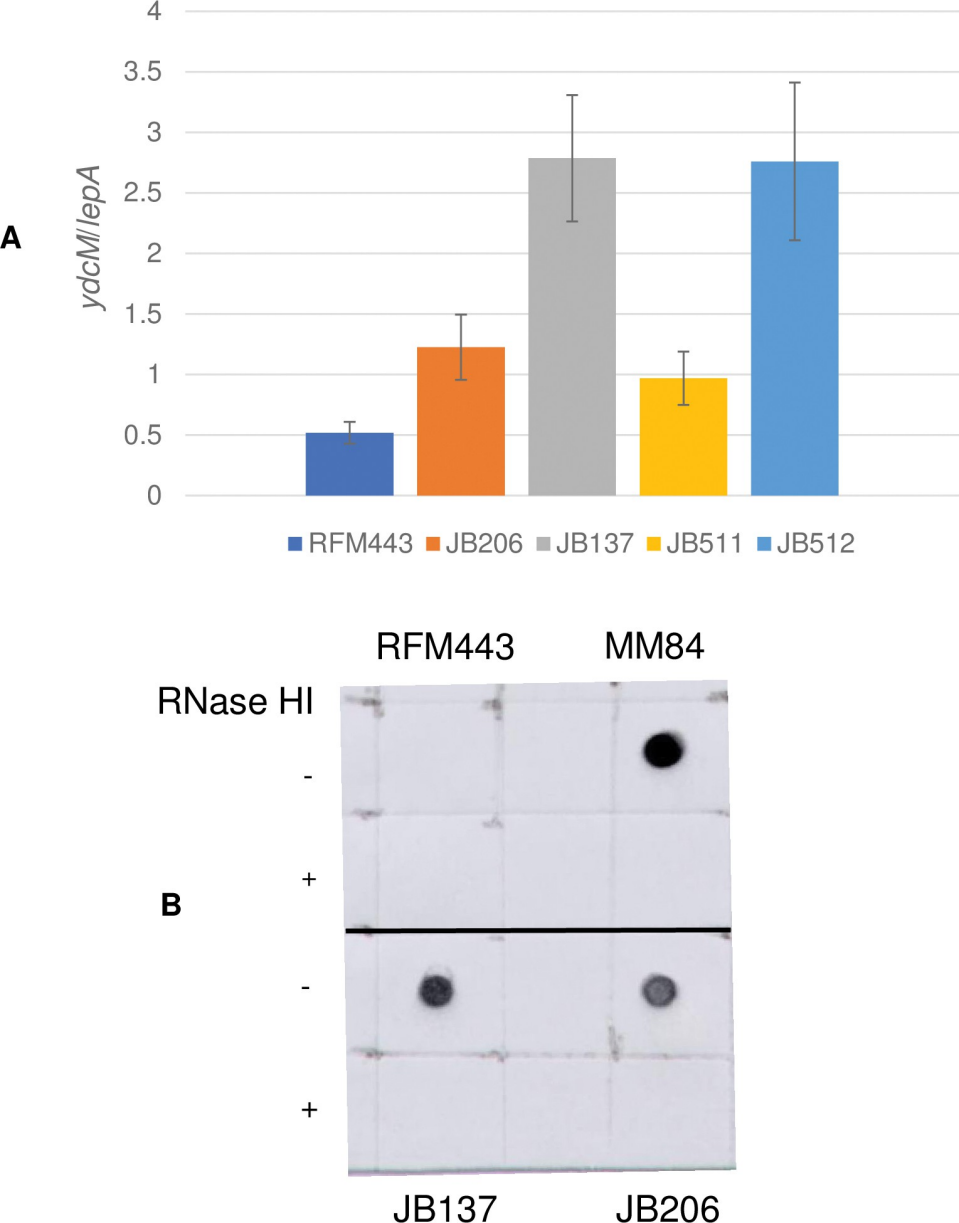
**The absence of *topB* in *topA* null cells dramatically increases the *ydcM*/*lepA* ratios (Ter peak height) but not R-loop formation.** (A) *ydcM/lepA* ratio determined by qPCR of genomic DNA extracted from RFM443 (wild-type), JB206 (*topA20*::Tn*10 gyrB*(Ts)), JB137 (*ΔtopB topA20*::Tn*10 gyrB*(Ts)), JB511 (*ΔtopB topA20*::Tn*10 gyrB*(Ts) pSK760), and JB512 (*ΔtopB topA20*::Tn*10 gyrB*(Ts) pSK762c) cells grown at 30°C to log phase as described in Materials and Methods. pSK760 but not pSK762c carries the wild-type *rnhA* gene to overproduce RNase HI. (B) Dot-blot with S9.6 antibodies of genomic DNA from RFM443 (wild-type), MM84 (*rnhA*::*cam*), JB137 (*ΔtopB topA20*::Tn*10 gyrB*(Ts)), and JB206 (*topA20*::Tn*10 gyrB*(Ts)) cells grown at 30°C. +RNase HI indicates that the genomic DNA was treated with RNase HI.

Although MFA data from our lab have previously shown that a Ter peak was present in single *topA* null strains, it was much less prominent than the Ter peak found in *topA topB* null strains [14]. Here, this is illustrated by the *ydcM/lepA* ratio showing that the Ter peak of the *topA* null strain is lower by 2.5 times than that of the *topA topB* null strain (Fig 5A, compare RFM443 (wild type), JB206 (*topA*) and JB137 (*topA topB*)), even though *topA topB* null cells grew slightly better than *topA* null cells at 30°C (Fig 4A). As previously demonstrated by MFA (), Fig 5A also shows that overproducing RNase HI significantly reduced the Ter peak height in *topA topB* null cells (*topA topB* cells overproducing (JB511) or not (JB512) RNase HI). This effect of RNase HI overproduction was shown to be mediated by the inhibition of R-loop formation that in turn inhibited RLDR [14]. Thus, we tested if the very strong increase in Ter peak height could be due to a dramatic increase in R-loop formation and RLDR when *topB* was also absent in *topA* null cells. A dot-blot experiment with S9.6 antibodies recognizing DNA:RNA hybrids was performed as previously to show R-loop formation in *topA topB* null cells [14]. As a control, the accumulation of R-loops in a *rnhA* null strain (MM84) but not in a wild-type strain (RM443) is shown in Fig 5B (Top left, RFM443 and right, MM84). Fig 5B also shows that R-loops accumulated only at a slightly higher level in *topA topB* null compared with *topA* null cells (bottom left, JB137 and right, JB206).

**Fig 6:**
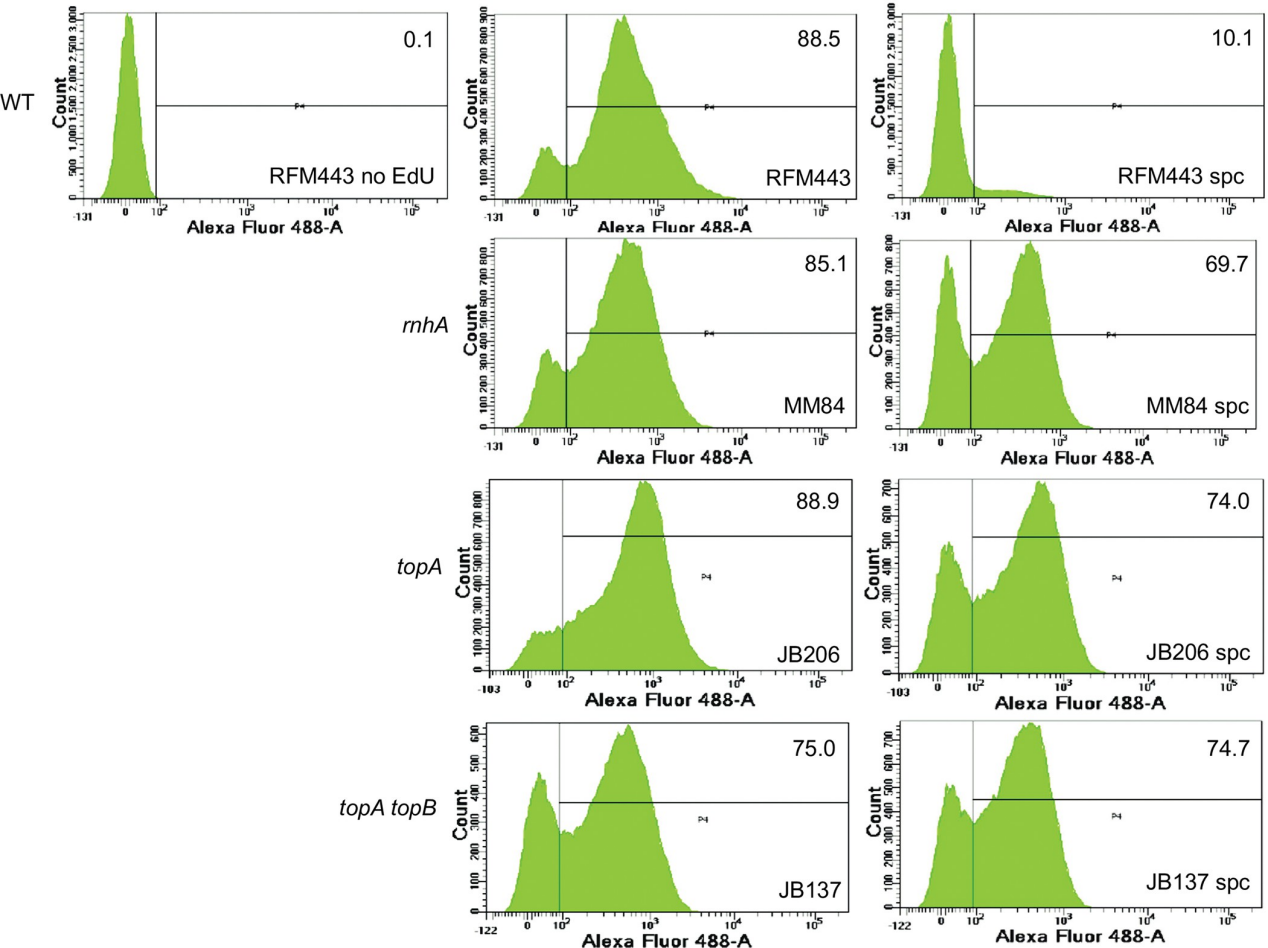
The absence of *topB* in *topA* null cells do not increase the level of RLDR. Flow cytometry to detect RLDR in RFM443 (wild-type), MM84 (*rnhA*::*cam*), JB206 (*topA20*::Tn*10 gyrB*(Ts)), and JB137 (*ΔtopB topA20*::Tn*10 gyrB*(Ts)) cells grown at 30°C as described in Material and Methods. The left panel is a no EdU control showing the position of the peak for cells not incorporating EdU (non-specific binding of the Alexa Fluor 448 dye). The middle panels are EdU but no spc, showing growing cells replicating DNA. The right panels (spc) are cells treated with spectinomycin for two hours before adding EdU to reveal RLDR. Numbers at the top right of the panels represent the percentage of cells incorporating EdU (replicating DNA).

A protocol to detect RLDR (cSDR) was developed in our lab [27,61]. This procedure allowed the detection of such RNase HI-sensitive DnaA-independent replication in both *topA* and *topA topB* null mutants. In this protocol, log phase cells are first treated with spectinomycin for two hours to inhibit the initiation of replication from *oriC* and to allow the already initiated DnaA-dependent replication to be terminated. EdU, a thymidine analog, is then added to the cells to detect RLDR. The cells are fixed after one hour, and the Alexa Fluor 488 dye is conjugated to EdU via the click chemistry. EdU incorporation is detected by flow cytometry. Fig 6, top, shows the results for the wild-type strain (RFM443). The left panel is the no-EdU control where the peak represents non-specific binding of the dye to the cells (this peak is on the left side of the vertical line in the diagram). A similar peak was seen for all the strains when EdU was not added to the cells, and this control is therefore not shown for the other strains. The middle panel represents cells incorporating EdU (no spectinomycin, cells replicating their DNA; the peak of replicating cells is on the right side of the vertical line in the diagram). The right panel shows the cells treated with spectinomycin for two hours before the addition of EdU. In the case of RFM443, the result demonstrates the absence of RLDR in wild-type cells. The numbers at the top right of the panels represent the percentage of cells replicating their DNA.

RLDR was readily detected in the *rnhA* null (MM84), *topA* null (JB206), and *topA topB* null (JB137) strains (Fig 6, spc, right panels). Importantly, levels of RLDR were very similar in *topA* null (JB206 spc, right panel, 74.0% of cells replicating their DNA) and *topA topB* null cells (JB137 spc, right panel, 74.7% of cells replicating their DNA). RLDR was also detected in *topA topB tus* (JB260) and *topA topB yncE IN(1*.*52–1*.*84)* (JB136) cells, as expected (S3 Fig). Thus, in agreement with the results of dot-blot experiments with S9.6, RLDR levels were found to be similar in *topA* (JB206) and *topA topB* null cells (JB137) and, therefore, could not explain the 2 to 3 times higher Ter peak in *topA topB* null cells compared with *topA* null cells. Thus, this increase in Ter peak height is most likely related to the function of topo III in decatenation during replication. This is supported by the data presented below.

### Topo IV overproduction corrects the chromosome segregation defects of *topA topB* null cells without reducing R-loop formation and RLDR

A decatenation problem related to the lack of a type IA topo activity could be compensated by an increased level of topo IV activity, to remove pre-catenanes during replication elongation and/or catenanes accumulating at the termination step. Indeed, topo III acts as a decatenase to eliminate pre-catenanes during replication, a function shared with topo IV, and may fully unlinks the parental DNA strands before replication termination, thus reducing the accumulation of catenanes at this step (Fig 1B). It has been previously shown that our *topA topB* null mutants carry an amplification in the region including the *parC* and *parE* genes, coding for the two subunits of topo IV [14]. This was evident in MFA profiles of *topA topB* null cells as shown in Fig 2A (JB137, labeled *amp*, genomic position 2.99–3.24). By measuring the *qseC*/*lepA* ratio by qPCR in which *qseC* is located in between *parC* and *parE* in the amplified DNA region, and *lepA* is close to, but outside the amplified region, it is possible to estimate the extent of *parC* and *parE* amplification. This procedure has been used previously and validated by MFA by NGS [14]. The *qseC*/*lepA* ratio is respectively 1.3 and 4.9 for the *topA* null and *topA topB* null strains (S4A Fig). Next, the *parC* and *parE* mRNA levels were measured by qRT-PCR with the use of *lepA* mRNA as a control. It was shown that the expression levels of *parC* and *parE* were 2- and 3-fold higher in *topA topB* null cells (JB137), respectively, compared with *topA* null cells (JB206) (S4B Fig).

**Fig 7:**
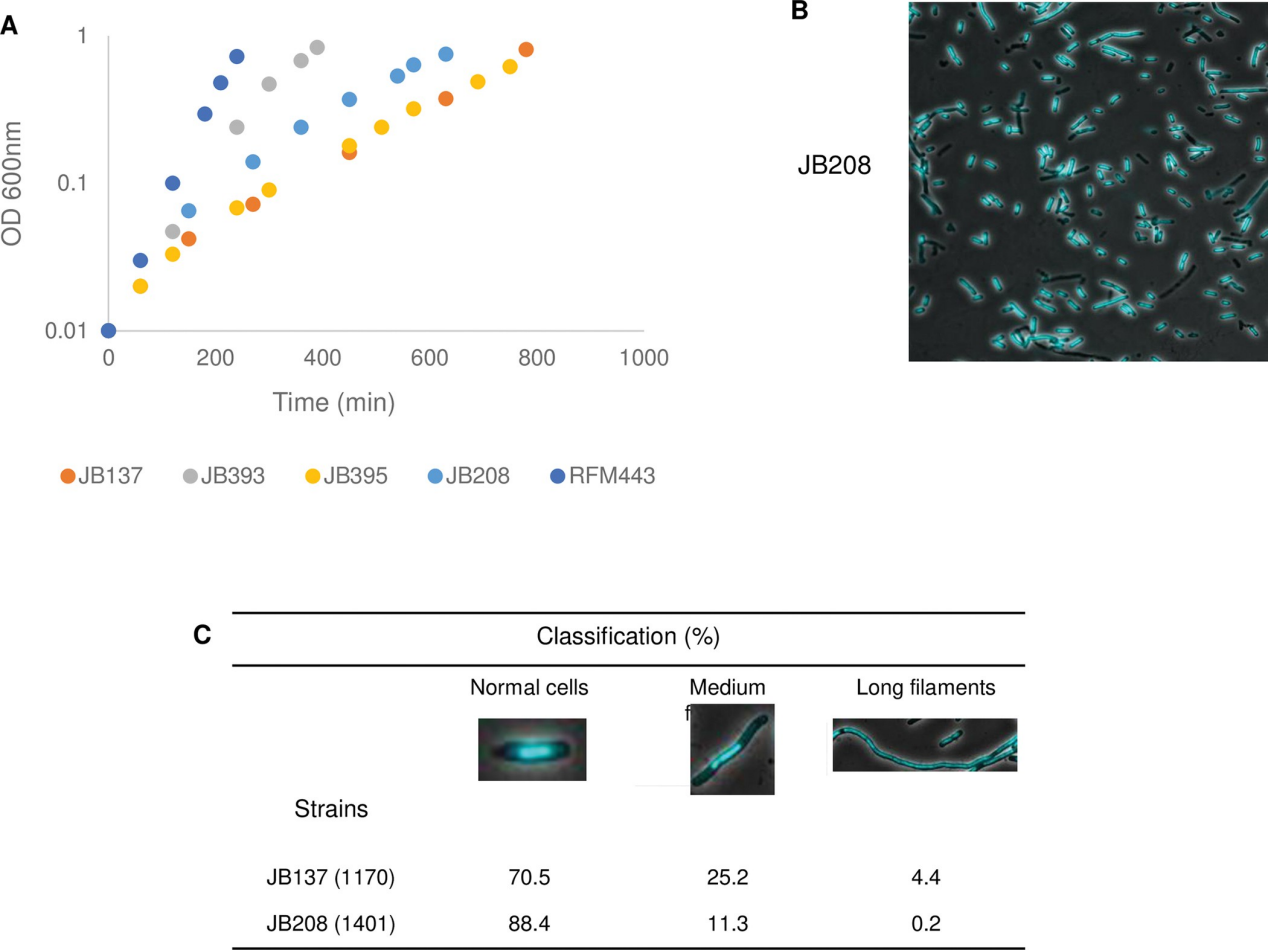
Increasing the level of topo IV overproduction with pET11-*parEC* slightly improves growth and significantly correct the filamentation and chromosome segregation defects of *topA topB* null cells. Cells of JB137 (*ΔtopB topA20*::Tn*10 gyrB*(Ts)), JB393 (JB137 pSK760), JB395 (JB137 pSK762c), JB208 (JB137 pET11-*parEC*) and RFM443 (wild-type) strains were grown overnight at 37°C on LB plates and diluted in fresh liquid LB medium for growth curve at 30°C (A) or for growth at 30°C to an OD_600_ of 0.8 for microscopy (JB208 only) (B), as described in Materials and Methods. A representative merged image of phase contrast and fluorescence pictures of SYTO-40-stained cells are shown in (B). In (C), cells (total number in parentheses) were examined in merged images to calculate the percentage of cells in each category. Results for JB137 were taken from Fig 4. pSK760 but not pSK762c carries the wild-type *rnhA* gene to overproduce RNase HI.

Despite the presumed 2 to 3-fold overproduction of Topo IV, the Ter peak was still very high in JB137 as shown in Fig 2. Thus, either topo IV had no effect on Ter or its expression level was still too low to significantly affect it. The JB137 strain was constructed at 37°C, a more permissive temperature than the 30°C, used in our experiments, and the level of topo IV amplification might be sufficient at 37°C but not at 30°C. The plasmid pET11-*parEC* was introduced in JB137 to increase the level of Topo IV overproduction. This plasmid produces a ParEC fusion protein that was shown to be active as a topo IV both *in vitro* and *in vivo* [62], and that could complement the growth defect of a *topA* null strain [37]. S4C Fig shows the results of qRT-PCR (*parC* and *parE*) for strain JB208 (*topA topB*/pET11-*parCE*) and strain JB137 (*topA topB*) for comparison. Considering that the fusion protein is 5- to 10-fold less active than the normal protein [62], the level of ParC and ParE activity overexpressed in JB208 are, respectively, approximately 3.5 and 5.5-fold compared to 2- and 3-fold for JB137 (S4C Fig, the adjusted overexpression levels are delineated by the black horizontal lanes for JB208). Thus, the level of topo IV activity is almost 2-fold higher in the *topA topB* null strain when pET11-*parCE* is present. Fig 7A shows that overproducing topo IV to this level (JB208, *topA topB*/pET11-*parCE*) improved the growth of the *topA topB* null strain (JB137), but not as well as overproducing RNase HI did (JB393, *topA topB*/pSK760). The cell morphology and chromosome segregation defects were very well corrected by the presence of the pET11-*parEC* plasmid in the *topA topB* null strain (compare the microscopy pictures of JB208, Fig 7B and JB137, Fig 4B and the quantitative data of JB208 and JB137, Fig 7C). Thus, overproducing topo IV to an appropriate level improved the growth of *topA topB* null cells and significantly corrected the chromosome segregation defect of these cells.

**Fig 8:**
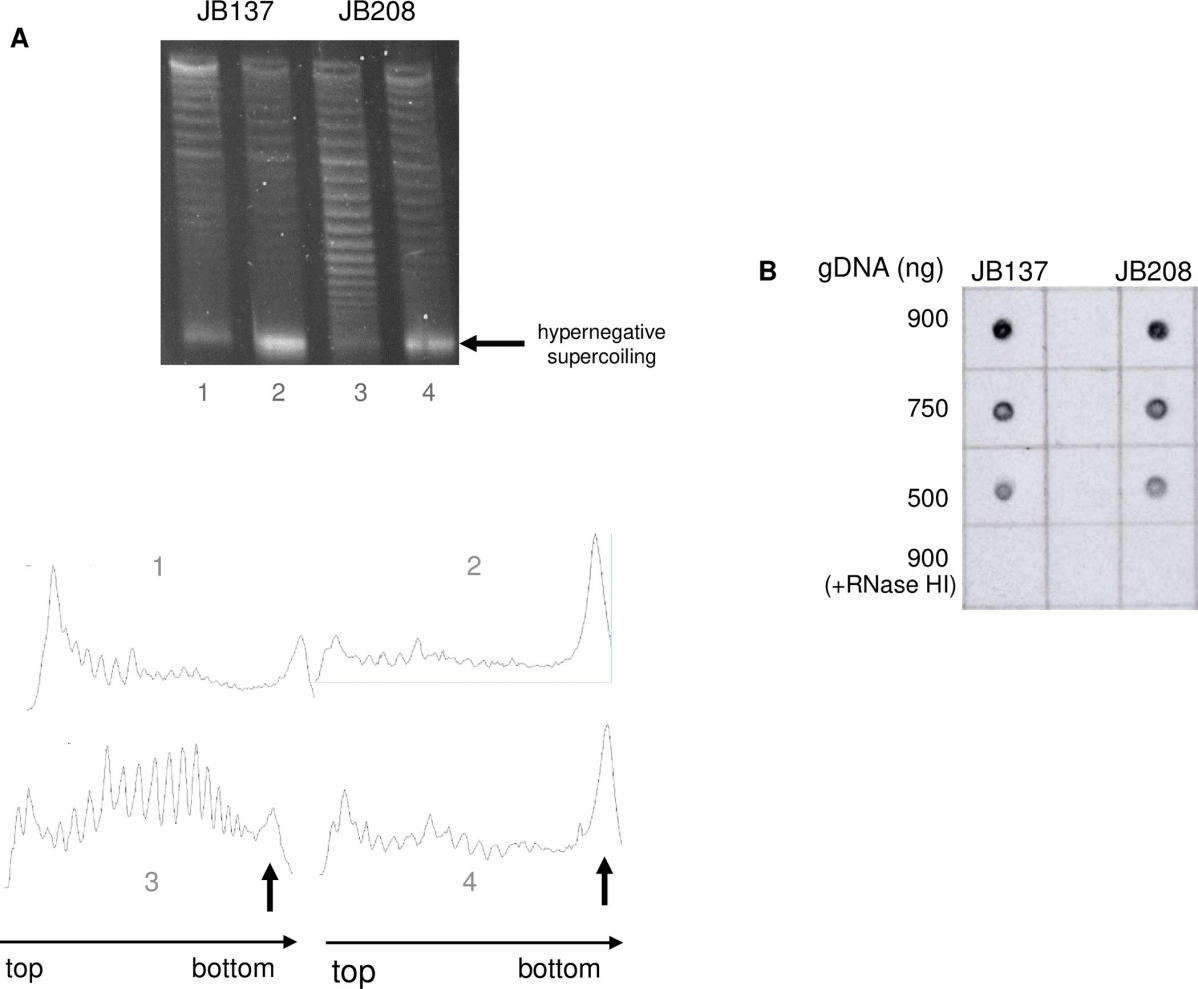
Increasing the level of topo IV overproduction with pET11-*parEC* slightly reduces hypernegative supercoiling but does not affect the level of R-loop formation in *topA topB* null cells. (A) One-dimensional chloroquine gel electrophoresis (7.5 μg/ml of chloroquine) of pACYC184Δtet5’ extracted from JB137 (*ΔtopB topA20*::Tn*10 gyrB*(Ts))/pACYC184Δtet5’ and JB208 (JB137 pET11-*parEC*)/pACYC184Δtet5’ cells grown at 37°C to an OD_600_ of 0.4 (lanes 1 and 3), or 30 min after a transfer from 37 to 30°C (lanes 2 and 4) as described in Materials and Methods. Arrows indicate hyper-negatively supercoiled DNA. Below is the densitometry tracing of the topoisomers in each lane. (B) Dot-blot with S9.6 antibodies of genomic DNA from JB137 (*ΔtopB topA20*::Tn*10 gyrB*(Ts)) and JB208 (JB137/pET11-*parEC*) cells grown at 30°C. The amount of genomic DNA spotted on the membrane is indicated. +RNase HI indicates that the genomic DNA was treated with RNase HI.

If topo IV corrected the chromosome segregation defect of *topA topB* null cells via its decatenation activity but not its relaxation activity on negative supercoiling, the data must show that topo IV had no significant effects on hypernegative supercoiling, R-loop formation, RLDR or the rate of Ter over-replication. This, indeed, is shown by the data presented below. The plasmid pACYC184Δ*tet*5’ was used to detect hypernegative supercoiling. This plasmid carries a deletion of the 5’ portion of the *tetA* gene that inactivates *tetA* gene translation [22]. This deletion inhibits hypernegative supercoiling in *topA* null mutants related to membrane anchorage of the transcription complex via the *tetA* gene product. Hypernegative supercoiling of this plasmid is linked to R-loop formation [22]. To detect hyper-negatively supercoiled plasmid DNA, electrophoresis in agarose gels in the presence of chloroquine at 7.5 μg/ml was performed. Under these conditions the more negatively supercoiled topoisomers migrate more slowly except for the fastest migrating band indicated by an arrow in Fig 8A (top panel), which represents hyper-negatively supercoiled plasmid DNA. Fig 8A (top, gel picture and bottom, densitometry analysis of the topoisomers) shows that overproducing topo IV from pET11-*parCE* had a significant negative effect (about 2-fold) on the level of hyper-negatively supercoiled DNA at 37°C, likely because gyrase supercoiling activity is reduced; in contrast, there was no significant negative effect at 30°C, the temperature re-activating gyrase that was also used in our experiments (37°C, compare lanes 1 (JB137) and 3 (JB208) and 30°C, lanes 2 (JB137) and 4 (JB208)).

**Fig 9:**
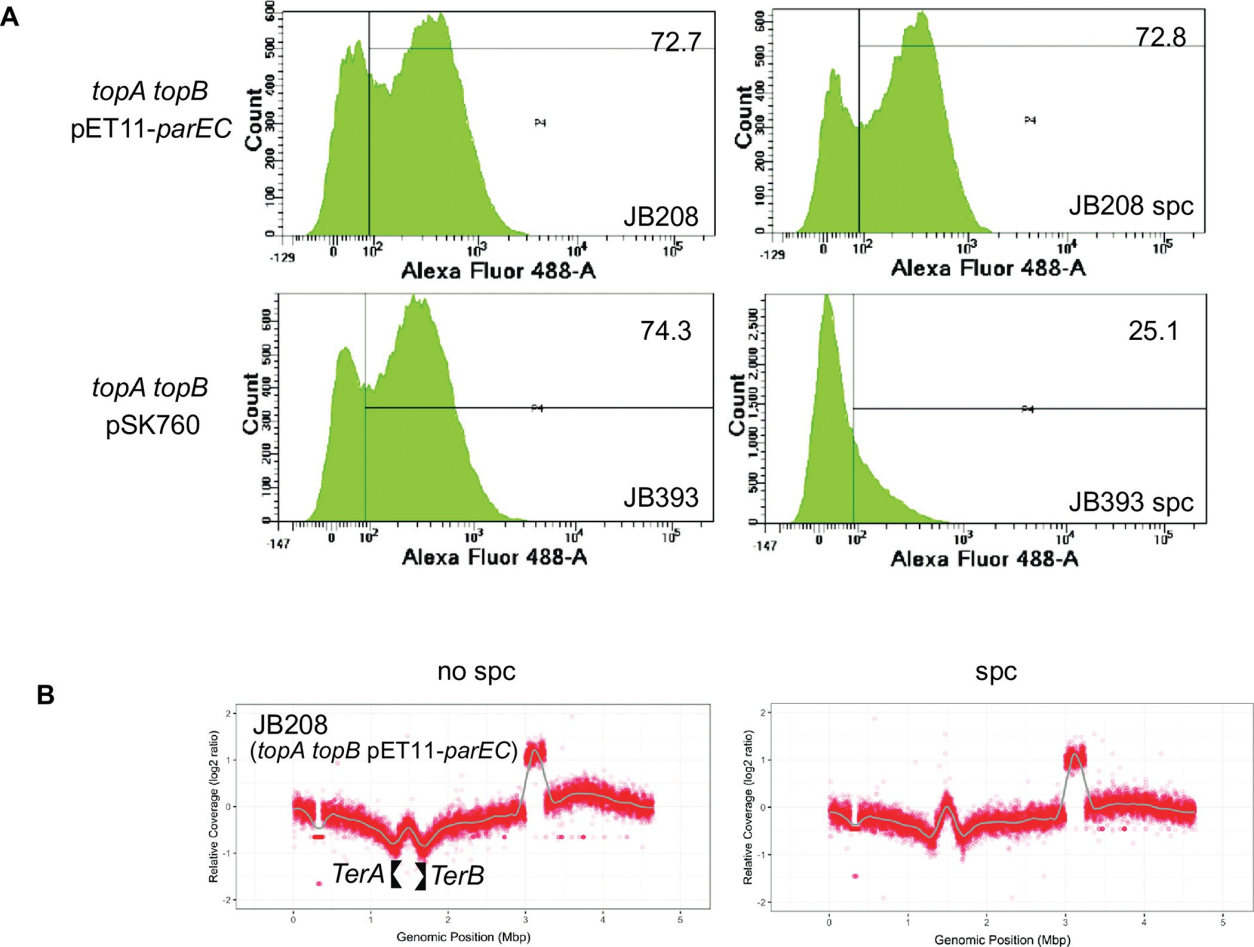
Increasing the level of topo IV overproduction with pET11-*parEC* has no effect on the level of RLDR but decreases the Ter peak height. (A) Flow cytometry to detect RLDR in JB208 (JB137/pET11-*parEC*) and JB393 (JB137 pSK760) cells grown at 30°C as described in Materials and Methods. pSK760 carries the wild-type *rnhA* gene to overproduce RNase HI. See the legend of Fig 6 for more details. (B) MFA by NGS of genomic DNA extracted from JB208 (JB137/pET11-*parEC*) cells grown at 30°C to log phase and treated (spc) or not treated (no spc) with spectinomycin for two hours. See the legend of Fig 2 for more details.

As expected from this result, Fig 8B shows that overproducing topo IV from pET11-*parCE* had no significant effect on the level of R-loop formation in *topA topB* null cells (dot-blot with S9.6, JB137 vs. JB208). In agreement with this result, the presence of pET11-*parCE* did not reduce the level of RDLR in *topA topB* null cells (Fig 9A, JB208 vs. JB208 spc compare with Fig 6, JB137 vs. JB137 spc). As a control, it is shown that overproducing RNase HI strongly inhibited RLDR in strain JB137 (Fig 9A, JB393 (JB137/pSK760), no spc vs. spc). The MFA profile of JB208 showed that the Ter peak height was reduced by approximately 2-fold when pET11-*parCE* was present in *topA topB* null cells (Fig 9B, JB208, compare with Fig 2A, JB137). The level of additional replication in the Ter region after the spectinomycin treatment was evaluated for strains JB137 and JB208, by determining the increase in the Ter peak height two hours after the addition of spectinomycin (see Materials and Methods). The values for JB137 and JB208 were found to be very similar, 0.234 and 0.210 respectively, despite the lower Ter peak for JB208. This suggested that the rate of DNA synthesis in the Ter region was very similar in the two strains, indicating that topo IV overproduction at a high level (JB208) acted via its decatenation activity by allowing chromosome segregation and then cell division. By doing so, cells were able to grow better, thus, there was more replication from *oriC*, explaining the Ter peak height reduction relative to the DNA copy number outside this region. Altogether, these results support the conclusion that the dramatic increase in Ter peak height when *topB* is absent in *topA* null mutants, is related to the lack of topo III decatenation activity during replication.

So far, the data have been obtained with strains carrying a *gyrB*(Ts) mutation and in the W3110 background. W3110 is one of the two extensively used *E*. *coli* K12 strains, MG1655 being the other one. W3110 carries an inversion of a long segment of approximately 783 kb originating from *rrnD* and *rrnE* operons that includes *oriC* [63]. In a previous study, *topA* null transductants were readily obtained in the MG1655 background and were thought to be viable without compensatory mutations [64]. One of these transductants was obtained, strain VS111 that has been used for the study of transcription-induced negative supercoiling [65–67]. The fact that this strain grew very well (S5A Fig, strain VS111, compared with RFM443, wild-type) may have indicated that it has acquired the frequent *parC parE* amplification. Indeed, the *qseC*/*lepA* ratio of VS111 was found to be 3.8 (S5B Fig), which demonstrated *parC parE* amplification in this strain. A *topB* null allele was introduced in VS111, and the resulting strain still grew well, but a bit slower than VS111 (S5A Fig, JB303, compared with VS111). This *topA topB* null strain (JB303) had a higher *qseC*/*lepA* ratio (S5B Fig, JB303, 6.8 and VS111, 3.6), consistent with the finding that *topA topB* null cells need more topo IV than *topA* null cells to grow [14]. This *qseC/lepA* ratio was higher than that of strain JB137 (S5B Fig, JB303, 6.8 vs. S4A Fig, JB137, 4.9) and led to a higher level of *parC* and *parE* mRNA overexpression as expected (qRT-PCR derived *parC/lepA* and *parE/lepA* ratios of 3.1 and 5.9 respectively for JB303 (S5B Fig), compared with 2 and 3 for JB137 and 3.5 and 5.5 for JB208 (S4B Fig). Thus, JB303 and JB208 (JB137/pET11-*parCE*) overproduced topo IV to similar levels.

The high level of topo IV overproduction likely explains, at least in part, the low Ter peak in JB303 (S5C Fig,—and + spc MFA profiles of JB303). Overproducing RNase HI from pSK760 had no effect on the growth of JB303, which already grew very well (S5A Fig, compare JB303, with JB350 (JB303/pSK760) and JB352 (JB303/pSK762c)). This MG1655 *topA topB* null strain accumulated R-loops (S5D Fig) and showed a high level of RLDR (S6 Fig, compare JB303 with JB350 and JB352). Thus, these results support those obtained with W3110 strains carrying a *gyrB*(Ts) allele (JB137) and clearly show that *topA topB* null strains overproducing topo IV to an optimal level, can grow well and tolerate a high level of R-loop formation and RLDR. This is consistent with the hypothesis that topological problems constitute a major cause of R-loop s toxicity.

### A strain carrying a naturally acquired compensatory gyrase mutation reduces RLDR and Ter over-replication in a *topA topB* null mutant

The effect of deleting *topB* on the *topA* null strain DM800 (W3110 background) carrying a naturally occurring compensatory *gyrB* mutation (*gyrB225*) was also studied. [9,10,55]. Here, a *topB* null derivative of DM800 was constructed (strain JB305) and its growth was found to be only slightly improved by overproducing RNase HI (S7A Fig, compare JB305, JB354 (JB305/pSK760) and JB356 (JB305/pSK762c)). JB305 grew slightly better than JB137 although more slowly than JB303 (JB305, S7A Fig, JB137, Fig 4A and JB303, S5A Fig). Deleting *topB* in DM800 significantly stimulated transcription-induced negative supercoiling. Indeed, whereas no hyper-negatively supercoiled pACYC184Δ*tet*5’ could be detected in DM800, such topoisomers were readily detected when *topB* was deleted (S7B Fig, compare lanes 1 and 2, DM800 with lanes 3 and 4, JB305, 37°C and 30°C; note that the *gyrB* mutation of DM800 is not thermo-sensitive). The stimulatory effect of the absence of *topB* in a *topA* null strain is also observed in strains with the *gyrB*(Ts) background at 30°C (S8 Fig. two-dimensional chloroquine gel, compare JB206 (*topA*) with JB137 (*topA topB*)). This suggests, as already proposed [14], that topo III can act as a backup for topo I, at least when topo I is absent. An alternative explanation is that when topo III is absent, more topo IV enzymes are required for decatenation during replication and therefore less are available to relax hyper-negatively supercoiled DNA.

RLDR was also detected in DM800 and JB305, although at a lower level than in the other *topA topB* null strains JB137 and JB303, consistent with the presence of the *gyrB225* allele that is also expressed at 30°C (S7C Fig, see JB354 (JB305/pSK760) and JB356 (JB305/pSK762c), and compare the low level of cSDR replication in DM800 with the higher one in *topA* null strains JB206 (Fig 6) and VS111 (S6 Fig)). The lower level of RLDR also led to a lower level of Ter over-replication compared with strain JB137 (S9 Fig. JB305, MFA profile–and + spc, compared with JB137, Fig 2A). S9 Fig also shows that a *parC parE* amplification was not detected in strain JB305. Thus, a naturally occurring gyrase mutation reduces enough RLDR to prevent the critical overloading of the decatenation capacity of topo IV in the absence of type IA topos.

### The rpoB*35 mutation making RNAP backtracking-resistant, reduces RLDR and Ter peak over-replication in a *topA topB* null mutant

Results from a previous study from our lab had suggested that RNAP mutations can affect R-loop formation in *topA* null mutants [68]. The *rpoB108* (*rpoB*: β subunit of RNAP) mutation was isolated, as a mutation conferring a stringent-like RNAP phenotype [68,69]. Stringent-like RNAPs compensate for the lack of ppGpp, the signalling molecule of the bacterial stringent response [70]. A *gyrB*(Ts) *topA* null mutant carrying this *rpoB108* mutation was shown to require less RNase HI activity to grow at 28°C than a *gyrB*(Ts) *topA* null mutant carrying the wild-type *rpoB* allele, which suggested that this mutation rendered RNAPs less prone to R-loop formation [68]. Supporting this hypothesis was the observation that the *rpoB108* mutation reduced R-loop-dependent hypernegative supercoiling of a plasmid [68]. In another study, the best-studied mutation conferring a stringent-like phenotype, rpoB*35, was shown to reduce transcription-replication conflicts [71] by reducing RNAP backtracking [72]. Furthermore, this rpoB*35 mutation, by reducing RNAP backtracking, inhibited R-loop formation associated with this process [72]. More recently, the rpoB*35 mutation was shown to allow *topA* null mutants to survive, although fast-growing colonies appeared on plates, suggesting the accumulation of *parC parE* amplifications [60].

**Fig 10:**
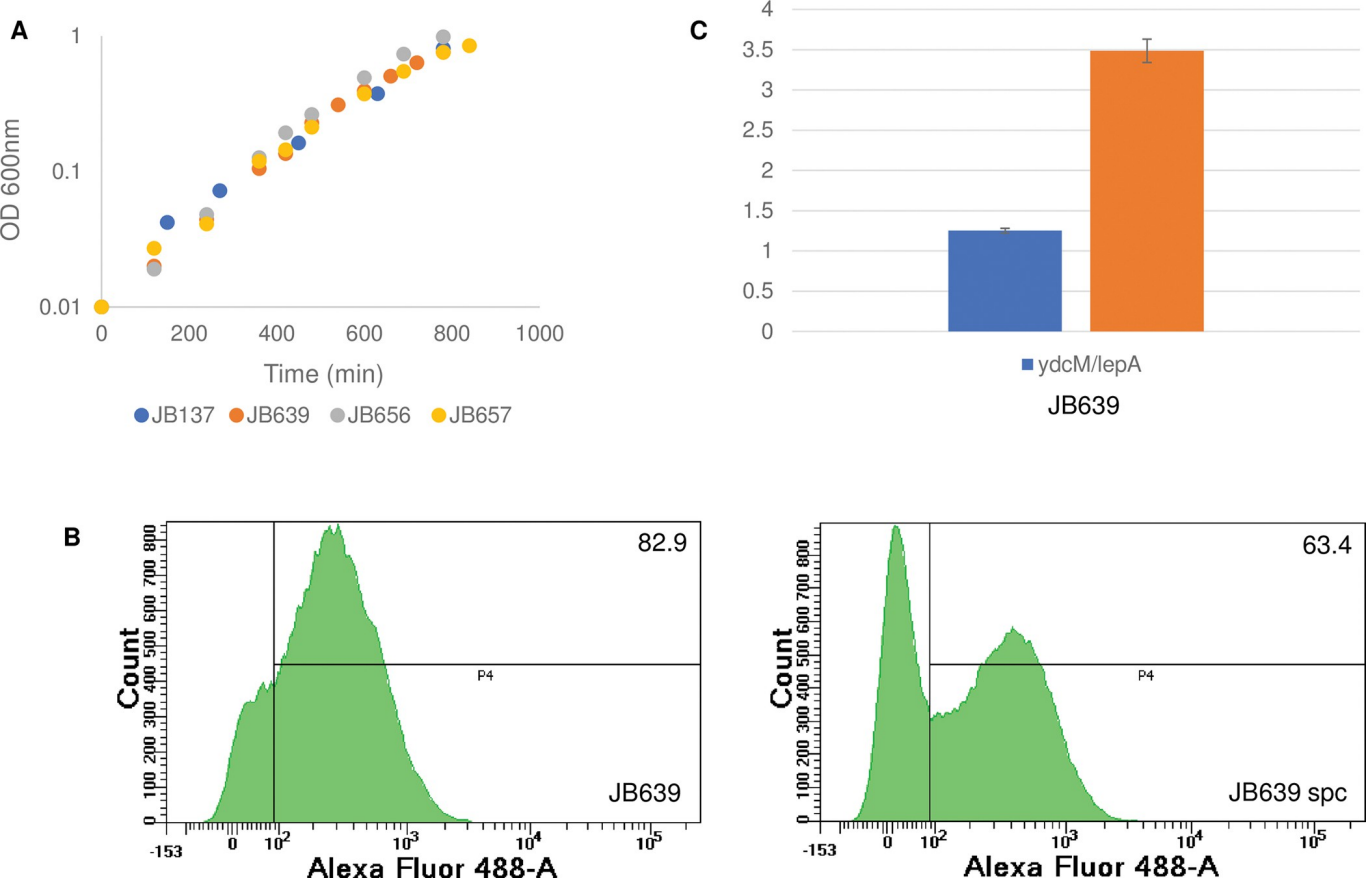
The rpoB*35 mutation inhibiting RNAP backtracking suppresses the positive effect of RNase HI overproduction on growth and reduces both the *ydcM/lepA* ratio and RLDR in *topA topB* null cells. (A) Cells of JB137 (*ΔtopB topA20*::Tn*10 gyrB*(Ts)), JB639 (*ΔtopB topA20*::Tn*10 gyrB*(Ts) rpo*35) JB656 (JB639 pSK760) and JB657 (JB639 pSK762c) strains were grown overnight at 37°C on LB plates and diluted in fresh liquid LB medium for growth curve at 30°C as described in Material and Methods. (B) Flow cytometry to detect RLDR in JB639 (*ΔtopB topA20*::Tn*10 gyrB*(Ts) rpo*35) cells grown at 30°C as described in Materials and Methods. See the legend of Fig 6 for more details. (C) *ydcM/lepA* and *qseC/lepA* ratios determined by qPCR of genomic DNA extracted from JB639 (*ΔtopB topA20*::Tn*10 gyrB*(Ts) rpo*35) cells grown at 30°C to log phase as described in Materials and Methods.

Here, effects of the rpoB*35 mutation on growth, cell morphology, RLDR and Ter over-replication in a *topA topB* null strain have been studied. Fig 10A shows that the presence of the rpoB*35 mutation did not affect the growth of *topA topB* null cells (JB137 (*topA topB*) vs. JB639 (topA topB rpoB*35)) and, importantly, unlike the case for *topA topB* null cells, RNase HI overproduction did not stimulate the growth of topA topB rpoB*35 cells (compare topA topB rpoB*35, JB639 (no plasmid), JB656 (pSK760) and JB657 (pSK762c)). Thus, similar to the situation described for the *rpoB108* mutation [68], these results suggested that the rpoB*35 mutation made the *topA topB* null cells less prone to the formation of toxic R-loops. This is supported by the results showing that rpoB*35 reduced both RLDR (Fig 10B, JB639, the + spc peak is clearly lower than the -spc peak, whereas both -spc and + spc peaks are similar for JB137, Fig 6) and the Ter peak height (Fig 10C, JB639, *ydcM/lepA* ratio of 1.2 compared with 2.8 for JB137, Fig 2B) in *topA topB* null cells. Fig 10C also shows that JB639 carried a *parC parE* amplification (JB639, *qseC/lepA* ratio of 3.5). The rpoB*35 mutation significantly corrected the filamentation and chromosome segregation defects of *topA topB* null cells (S10A Fig, qualitative and S10B Fig, quantitative results). Thus, these results suggested that RNAP backtracking is, at least partly, responsible for the formation of toxic R-loops leading to RLDR in *topA topB* null mutants.

### Complementation of the RLDR phenotype of *topA topB* null mutants by truncated versions of *E*. *coli* topo I and by topo I from *Mycobacterium* species

The catalytic N-terminal domain for the cleavage and rejoining of ssDNA/RNA is highly conserved in type IA topos [73]. The C-terminal domain that is required for processive DNA relaxation is less conserved and confers various capabilities for DNA/RNA and protein interactions that have important consequences for their physiological functions [73]. Here, experiments were performed to verify the requirement of an intact *E*. *coli* topo I C-terminal domain to inhibit RLDR in *E*. *coli topA topB* null mutants. S11 Fig shows that the full-length topo I (pJW312, JB501 spc) as well as an 85 kDa derivative that lack the 14-kDa C-terminal fragment of topo I (Top85, pJW2277, JB503, spc) efficiently inhibited RLDR in JB303strain (top, MG1655 *topA topB*). Previously, it has been shown that Top85 had 75% relaxation activity and reduced processivity, complemented the growth of a *topA* null mutant, and was 100 times more sensitive than the full-length topo I to killing by the toxic electrophile *N*-ethylmaleimide (NEM) [20]. The 67 kDa N-terminal domain that has the active site tyrosine for DNA cleavage but cannot relax negative supercoiling (Top67, pJW67, JB505 spc) was unable to inhibit RLDR (S11 Fig).

Next, the ability of *Mycobacterium smegmatis* and *Mycobacterium tuberculosis* topo I to inhibit RLDR in the *E*. *coli topA topB* null mutant JB137 was tested. These topos have a different C-terminal region, Topo_C_Rpt without cysteines for *Mycobacterium* topo I compared with *E*. *coli* topo I, Topo_C_ZnRpt containing C4 zinc finger [73]. Despite these different C-terminal domains, both *M*. *smegmatis* and *M*. *tuberculosis* significantly inhibited RLDR in *E*. *coli* JB137 strain (S12 Fig,—and + spc, JB137, JB472 (JB137/p2OT-MOCR, empty vector), JB456 (JB137/p2OT-Msmtop, *M*. *smegmatis* topo I), and JB458 (JB137/p2OT-Mtbtop, *M*. *tuberculosis* topo I)). Altogether, these results revealed that the negative supercoiling relaxation activity without specific topo I-protein interactions mediated by the C-terminal domain, appears to be sufficient for a type IA topo to inhibit RLDR in *E*. *coli topA topB* null mutants. However, since the actual level of Msm and Mtb topo I proteins produced from the plasmids is not known, it is not possible to compare the efficiency of these topos I with that of *E*. *coli* topo I in inhibiting RLDR in *E*. *coli*.

## Discussion

A genetic screen for mutations allowing *topA rnhA* null cells to survive has previously revealed that RLDR was a major source of R-loops toxicity in *E*. *coli* [28,74]. However, these results did not reveal the nature of RLDR, the mechanism(s) responsible for its toxicity, or how the cell can address it. More recently, the possible presence of origin(s) for RLDR (*oriKs*) in Ter was evoked to explain over-replication in this region and it was proposed that this replication was responsible for the R-loop-dependent phenotypes of *topA topB* null cells [14]. However, the recent demonstration that Ter over-replication in various mutants for genes involved in DNA replication and recombination, including *rnhA*, was not due to the presence of replication origins in this region but presumably to replication termination problems (see Introduction), led us to further study Ter over-replication in *topA topB* null cells.

Here, it was shown that: 1) Deleting genes located at the top of the Ter peak, including one associated with a high level of R-loop formation in *topA topB* null cells, did not significantly reduced the Ter peak height, suggesting that strong *oriKs* are not present in this region. 2) Deleting *tus* generated a flattened MFA profile thus confirming the absence of strong well-defined origins for RLDR (*oriKs*) in Ter as well as outside this region and suggesting that *Ter*/Tus barriers are involved in over-replication. Furthermore, this result suggested, as previously proposed, that many weak origins for RLDR widely distributed across the genome, stochastically generate replication forks in the population of cells (and see below) 3) Switching the orientation of the *TerB*/Tus barrier re-localized the over-replicated region, now spanning more than 1 Mbp in length, to the right side of *TerB*, strongly suggesting that this barrier is involved in over-replication. 4) The major increase in Ter peak height upon deleting *topB* in *topA* null cells was not due to an increase in R-loop formation and RLDR, implying that it was instead related to the lack of topo III decatenation activity during replication. 5) Overproducing topo IV, the main cellular decatenase, did not correct R-loop and RLDR phenotypes, but corrected the chromosome segregation defect associated with Ter over-replication in *topA topB* null cells. 6) The finding that the rpoB*35 mutation making RNAP resistant to backtracking reduced RLDR and Ter over-replication suggested that RLDR could be triggered by the formation of R-loops involving RNAP backtracking. Below, a model is presented that explains the roles of type IA topos in replication termination, and possible mechanism(s) for the origin of RLDR are discussed in more detail.

### The involvement on type IA topos in replication termination

Previous data have shown that Ter over-replication was clearly linked to RLDR and that it was initiated by the absence of *topA* but not *topB* [14]. The presence of the small Ter peak in *topA* null cells could simply reflect the presence of many weak origins for RLDR widely distributed across the genome and stochastically generating replication forks in the population of cells, that are arrested at the innermost *Ter/*Tus barriers. However, our data suggest that it was the accumulation of forks *per se* at these *Ter*/Tus barriers that triggered the whole process, as the very high Ter peak was not observed when *tus* was absent in *topA topB* mutants. Importantly, in a strain with an inverted *TerB*/Tus barrier, the over-replicated region was re-localized at the right side of *TerB*, as expected if forks trapped at the *TerB*/Tus barrier triggered this over-replication. Under normal conditions, when convergent forks are initiated from *oriC*, their processing at the opposite *Ter*/Tus barriers prevents over-replication [38]. In this situation, the dwell time for a replication fork at a *Ter*/Tus barrier before the arrival of the convergent fork, is short. However, when ectopic origins of replication are active, i.e., RLDR in *topA* mutants, this dwell time might be much longer. In this situation, *recA*-dependent Ter over-replication has been observed in wild-type cells [52] and in *recG* mutants [44], although much more in the latter. Ter over-replication in both *topA* and *topA topB* null mutants is also RecA-dependent [14]. Moreover, fork-processing at *Ter*/Tus barriers has been shown to be defective in *topA* mutants due to excess negative supercoiling [53]. We propose that in *topA* mutants, the increased dwell time of forks at *Ter*/Tus barriers due to RLDR and the defective processing of these forks due to excess negative supercoiling, saturate the convergent fork-processing machinery including RecG. In this situation, Ter over-replication is observed.

The Ter peak height in *topA* null mutants considerably increased upon *topB* deletion due to the lack of type IA topos activity in decatenation. Here, we envision two mechanisms by which the lack of type 1A topo decatenation activity can affect the Ter peak height. The first, directly affects Ter over-replication and can only be prevented by topos of the type IA family, whereas the second acts after Ter over-replication to increase the Ter peak height and can be prevented by topo IV overproduction. In the first mechanism, the defective processing of forks at *Ter*/Tus barrier is *topA* mutants leading to the saturation of the convergent fork-processing machinery (see above), is further aggravated by the lack of topo III activity that fully eliminates pathway 2 for resolving the topological problems of forks fusion (Fig 1B). Indeed, the use of pathway 2 involving a type IA topo for termination would prevent pathological fork fusion by fully unlinking the parental DNA strands before the completion of replication. In this model, replication (gap filling) can be completed by polI (*polA*). Interestingly, over-replication in *Ter* has been observed in *polA* mutants [49]. However, more work is still needed to fully understand the interplay between *Ter*/Tus barriers, Ter over-replication and type IA topos.

In the second mechanism, the chromosome segregation defect combined with the continuous *Ter* over-replication led to the dramatic increase in Ter peak height. Recently, it was shown that the failure to remove pre-catenanes during replication might render the process of fork convergence very inefficient by hindering fork rotation, a prerequisite for termination via pathway 1 (Fig 1B), thus causing the accumulation of positive supercoiling that significantly delay fork convergence [75]. This problem would be exacerbated in *topA topB* null mutants, because of the lack of topo III during replication to remove pre-catenanes and the absence of type IA topos in termination (pathway 2, Fig 1B). Thus, the lack type 1A will not only promote Ter over-replication but will also lead to the accumulation of catenanes in a small chromosomal region (Ter) that will significantly delay chromosome segregation, unless topo IV is overproduced.

### The mechanism of complementation of type IA topos mutants by overproducing Topo IV

Interestingly, topo IV overproduction to an appropriate level, significantly corrected the chromosome segregation and growth defects of *topA topB* null mutants without correcting R-loop formation and RLDR and by reducing only slightly hypernegative supercoiling. The positive effects of topo IV were particularly very strong in the strain in which topo IV overproduction to the appropriate level occurred via a naturally selected *parC parE* amplification (strain JB303). So, topo IV overproduction allows type IA topos mutants to tolerate high levels of R-loop formation by correcting the downstream effects: topological problems due to RLDR, corrected via decatenation, and R-loop-induced hypernegative supercoiling [76,77], corrected via relaxation. The beneficial effects of topo IV overproduction on single *topA* null mutants may also be exerted through similar decatenation and relaxation activities.

### RLDR mechanism(s)

**Fig 11:**
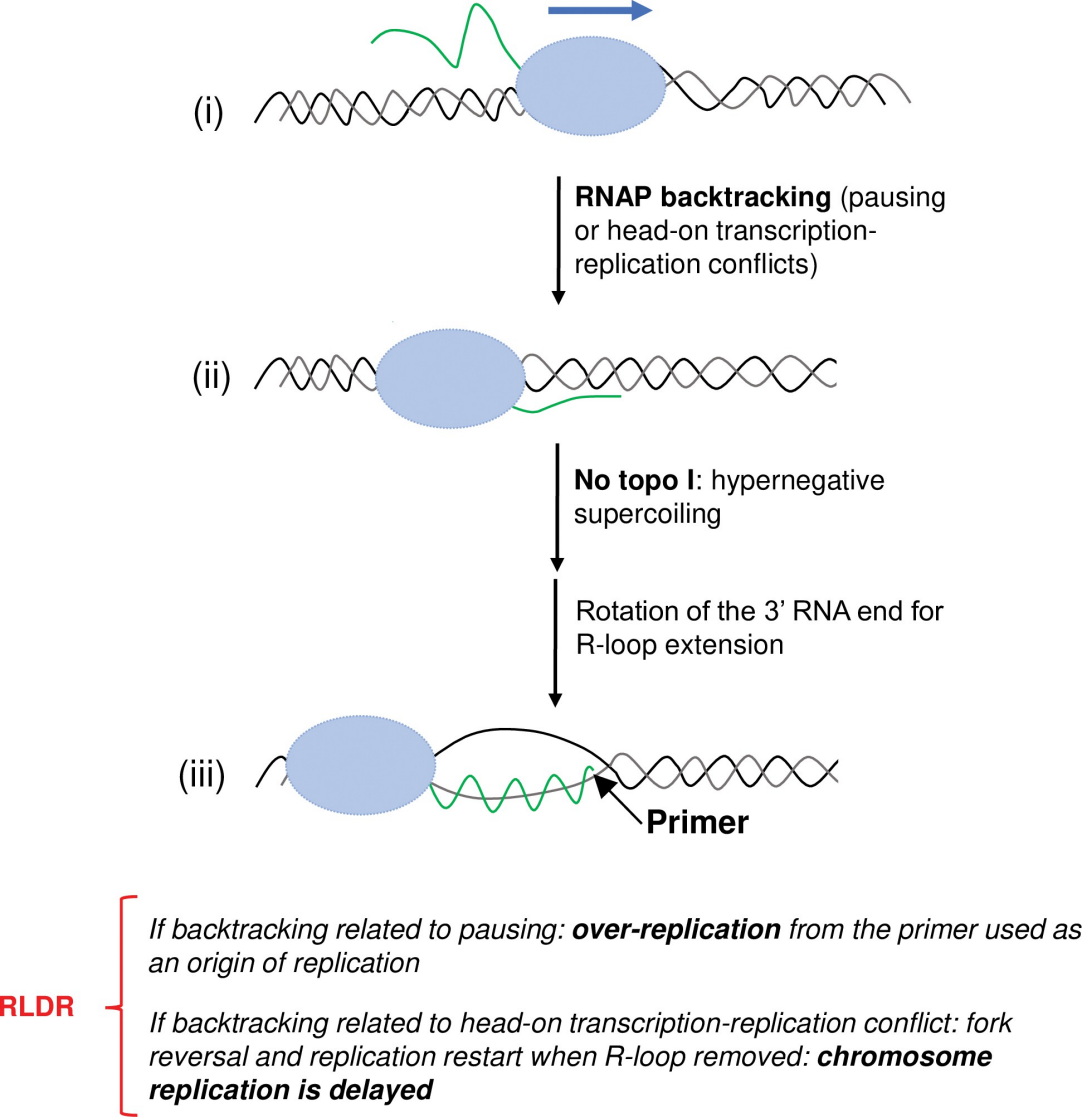
Model for the effect of RNAP backtracking on RLDR in the absence of topo I. RNAP pausing is known to induce RNAP backtracking (ii). In the absence of topo I, hypernegative supercoiling may lead to the annealing of the free RNA to the template strand in front of the backtracked RNAP (iii). This leads to the formation of an R-loop with a free 3’ RNA end available to prime replication (over-replication) that may contribute to the accumulation of replication forks at *Ter/Tus* barriers. RNAP backtracking following head-on collisions between transcription (e.g., *rrn* operons) and replication (e.g., initiated from R-loops and in the wrong orientation (toward *oriC*)) can lead to R-loop formation that efficiently blocks replication, thus delaying the completion of chromosome replication. See text for details.

Here, the data showed that the rpoB*35 mutation making RNAP resistant to backtracking and conferring a stringent-like RNAP phenotype, reduced RLDR and Ter over-replication, and corrected the chromosome segregation defect of *topA topB* null cells. Moreover, RNase HI overproduction had no effects on the growth of *topA topB* null cells carrying this mutation. Previously, this rpoB*35 mutation, by reducing RNAP backtracking, was found to inhibit R-loop formation associated with this process [72]. Interestingly, in this previous study, the R-loops generated by RNAP backtracking could be used as primers for replication. In another study, the rpoB*35 mutation was shown to reduce transcription-replication conflicts by reducing RNAP backtracking [41,71,72]. More recently, the rpoB*35 mutation could partially compensate for the growth defect of *topA* null mutants [60]. This result agrees with the previous finding that the *rpoB108* allele, a mutation also conferring a stringent-like RNAP phenotype, partially corrected the growth defect of a *topA* null mutant and reduced R-loop-dependent hypernegative supercoiling [68]. Altogether, these results suggest that a significant fraction of toxic R-loops in *topA* null cells, those that are somehow involved in replication, originate from RNAP backtracking. We propose that the hypernegative supercoiling generated in the absence of topo I can promote RNAP backtracking and the annealing of the RNA with the DNA template strand during this process (Fig 11). Gyrase activity can also promote R-loop formation via this process as suggested by the finding reported in this work that a naturally occurring *gyrB* mutation (*gyrB225*), significantly reduced RLDR and Ter over-replication in a *topA topB* null mutant.

R-loop formation generated by backtracking unlike R-loop formation generated behind the moving RNAP, generates a free RNA 3’ end that is available to initiate replication (Fig 11). Indeed, in the case of R-loop formation behind the RNAP, the RNA 3’ end in the RNAP transcription bubble is unavailable to initiate replication, unless this situation also leads to RNAP backtracking with the generation of an RNA:DNA hybrid with a free RNA 3’ end. Thus, we suggest that RNAP backtracking-mediated R-loop formation is at least partly responsible for the stochastic nature of RLDR initiation at many different sites on the chromosome in *topA topB* null mutants. However, RLDR may not always be the result of newly initiated replication as it may sometimes be due to replication restart. Indeed, head-on replication-transcription conflicts involving R-loop formation have been shown to stably arrest replication forks that may re-start once the R-loop is removed [78]. Head-on replication conflicts in cells lacking type IA topos may be triggered by new replication (RLDR) initiated in the reverse orientation relative to replication from *oriC*, which may lead to conflicts with the heavily transcribed *rrn* operons as previously shown [43]. Thus, both RLDR via newly initiated replication and via replication restart may contribute to the observation that a high level of DNA synthesis is still detected more than two hours after the addition of spectinomycin to inhibit replication initiation from *oriC* in *topA topB* cell cultures. Here, it was also shown that RLDR in our *E*. *coli topA topB* null mutants could be inhibited by *Mycobacterium* topos I, which have very different C-terminal regions relative to *E*. *coli* topo I. Thus, it is probable that *Mycobacterium* topo I do not interact with *E*. *coli* RNAP, suggesting that this interaction is not required for the inhibition of backtracking-mediated R-loop formation.

## Conclusion

Here, a pathway for genomic instability in cells lacking type IA topos has been described: RLDR involving RNAP backtracking, causes the accumulation of replication forks at *Ter/*Tus barriers that triggers over-replication leading to a chromosome segregation defect. RLDR is the result of the lack of topo I activity to remove transcription-induced negative supercoiling but can be further stimulated by deleting *topB* in *topA* null mutants. A high level of Ter over-replication and the related chromosome segregation defects require that both topo I and III activity be absent, with topo I possibly acting as a backup for topo III. Both the inhibition of R-loop formation and of *Ter*/Tus-dependent over-replication are unique functions of topos from the type IA family. Gyrase, by stimulating RLDR via its supercoiling activity, exacerbates R-loop-dependent genomic instability, whereas topo IV, by acting as a decatenase, alleviates it.

## Materials and methods

### Bacterial strains and plasmids

The list of bacterial strains used in this study as well as the details of their constructions can be found in S1 Table. This table also provides the list of plasmids used in this work. pET His6 Mocr TEV LIC cloning vector (2O-T) was a gift from Scott Gradia (Addgene plasmid # 29710; http://n2t.net/addgene:29710; RRID:Addgene_29710). Plasmids p2OT-Msmtop, p2OT-Mtbtop, pJW312, pJW67 and pJW2277 were gifts from Yuk-Ching Tse-Dinh, Florida International University. Transductions with phage P1*vir* were done as described previously [37]. PCR with appropriate oligonucleotides, and sequencing when needed, were performed to confirm the transfer of the expected alleles in the selected transductants. The inversion in JB136 occurred naturally and involved recombination between short homologous sequences (FRT, FLP recognition target) in the *yncE* and the *topB* loci that are the result of FLP recombinase activity to remove the Km^R^ cassette [79] that was introduced to substitute for these two genes during the process of JB121 strain construction. Therefore, the inversion in JB136 was named *IN(1*.*52–1*.*84)*.

### Bacterial cells growth

Cells were grown overnight at 37°C on LB plates with the appropriate antibiotics and supplements added. Cell suspensions were prepared, and aliquots were used to obtain an OD_600_ of 0.01 in fresh LB media. The cells were grown at the specified temperature to log phase at the indicated OD_600_.

### Plasmid extraction for supercoiling analysis

pACYC184Δ*tet*5’ DNA extraction for supercoiling analysis was performed by using the Monarch Plasmid Miniprep Kit (NEB). Cells were grown at the indicated temperature to an OD_600_ of 0.4. We found that after the first centrifugation step, keeping the cell pellet on ice for 30 minutes instead of resuspending it immediately in the plasmid resuspension buffer did not affect the results. Chloroquine gel electrophoresis (7.5 μg/ml chloroquine) was performed as previously reported [77].

### Detection of RLDR

For the detection RLDR, EdU (ethynyl deoxyuridine) incorporation and click-labelling using the “Click-It Alexa Fluor 448 Imaging kit” (Life Technologies, Molecular Probes) were conducted as previously described [27,61]. Briefly, the cells were grown in LB medium at 30°C to an OD_600_ of 0.3. An aliquot of cells was used for EdU incorporation for 60 min to detect ongoing replication in log phase cells. To detect RLDR, the log phase cells were first treated with spectinomycin for two hours, to allow the termination of replication rounds initiated from *oriC*, before EdU incorporation for 60 min. After click-labelling, replication (EdU incorporation) was detected by flow cytometry using a FACS Canto II apparatus (BD), and the data were analyzed and generated using the FACS Diva software. The apparatus was set to read 50 000 cells per experiment. Upon repeating experiments with identical parameters (growth and cell numbers) the results were found to be highly reproducible. The presence of control plasmids in strains did not affect the results.

### MFA by NGS

MFA by NGS was performed essentially as previously described [14]. Cells were grown at 30°C to an OD_600_ of 0.4. An aliquot of 10 ml of cell culture was transferred in a tube filled with ice for genomic DNA extraction. Spectinomycin (400 μg/ml) was added to the remaining cell culture and the cells were incubated for an additional two hours at the same temperature; an aliquot of 10 ml of cell culture was then taken and treated as above. Cells were recovered by centrifugation. Genomic DNA was extracted by using the GenElute bacterial genomic DNA kit (Sigma Aldrich) as described by the manufacturer except that the treatment to proteinase K was for two hours instead of 30 min. This modification eliminates artefacts in the MFA profiles around the *rrn* operons [80]. Sequencing was performed by using Illumina NovaSeq 6000 (Génome Québec, Montréal, Canada) to determine the sequence copy number. Bioinformatics analysis was performed at the Canadian Centre for Computational Genomic (C3G, McGill University, Montréal, Canada). The *E*. *coli* K12 W3110 genomic sequence AP009048.1 was used as the reference for the read mapping (15 to 20 million sequencing reads per sample). To reduce miscalculation of depth of coverage due to reads mapping at multiple places in the genome, a minimum mapping quality of 10 (phred scale based) was used for a read to be kept during the calculation of the depth of sequencing. The number of reads was normalized against a stationary phase wild-type control to consider differences in read depth across the genome of cells. Enrichment in 500 bp windows (on average) across the genome (10,000 points) was calculated, and loess regression curves were generated with loess-span parameters set to 0.1. To evaluate the level of additional replication in the chromosomal Ter region after the spectinomycin treatment, we first determined the difference between the highest loess value in the Ter peak and the lowest value on the right side of the peak before and after the addition of spectinomycin. We then subtracted the number obtained for the MFA profile before spectinomycin to the one after the treatment.

### qPCR

The genomic DNA for qPCR was prepared as described above for NGS. The Maxima SYBR Green qPCR Master Mix (2X) (ThermoFisher Scientific) was used with a Rotor-Gene 6000 (Corbett) apparatus. For each experiment two tubes were prepared that contained 50 and 100 ng of genomic DNA, respectively. Experiments were repeated at least twice for each set of primers. The ratios were determined by using the 2^-Δct^ formula and standard deviations were calculated from these values. The primers were designed by using the PrimerQuest tool (IDT). Forward and reverse primer sequences (5’-3’) were GAGTACCGGGCAGACCTATAA and AGCCTACTTCGCCACATTTC for *lepA*, CTGGACTCACTGGATAACCTTC and TGCGCCGTGTGGTAAATA for *qseC*, and CGAGACTTCAGCGACAGTTAAG and CCTGCGGATATTTGCGATACA for *ydcM*.

### Fluorescence microscopy

Cells were grown to an OD_600_ of 0.8, centrifuged and resuspended in 1X PBS. Cells were mixed with polyformaldehyde 4% and a 2 μl aliquot was spread on a coverslip previously treated with (3-aminopropyl)triethoxysilane. Two μl of a 1/1000 dilution of SYTO 40 (ThermoFisher Scientific) was applied to the dried samples and the coverslip was deposited on a slide. Microscopy was performed by using a Nikon Ti2 apparatus with a Plan Apochromat 1.45 NA oil immersion 100x phase contrast objective and CFP filter set for epifluorescence imaging. The camera was a Hamamatsu Orca Flash 4.0. Images were processed by using ImageJ software.

### Dot blots with S9.6 antibodies

Genomic DNA for the dot blots with S9.6 antibodies was prepared as described above for NGS, except that the amount of RNase A added was reduced by 20-fold. Treatment of the genomic DNA to RNase III or RNase HI, as well as dot blotting were performed as described previously [14,81].

## Supporting information

S1 Fig DRIP-qPCR showing R-loop formation at the *ydcD* locus in *topA topB* null cells. Cells of MM62 (wild-type), MM84 (*rnhA*::*cam*) and VU425 (*ΔtopB topA20*::Tn*10 gyrB*(Ts)/pSK762c (Usongo V, Drolet M. (2014) Roles of type 1A topoisomerases in genome maintenance in *Escherichia coli*. PLoS Genet. 2014;10(8):e1004543.) strains were grown overnight at 37°C on LB plates and diluted in fresh liquid LB medium for growth at 30°C to an OD_600_ of 0.4. Genomic DNA was extracted by using the GenElute bacterial genomic DNA kit (Sigma Aldrich) as described by the manufacturer, except that 1 μl of RNase A instead of 20 was used. DRIP (DNA-RNA immunoprecipitation)-qPCR was performed exactly as described in Sanz and Chédin (Sanz LA, Chédin, F. (2019) High-resolution, strand-specific R-loop mapping via S9.6-based DNA-RNA immunoprecipitation and high-throughput sequencing. Nat Protoc. 2019 Jun;14(6):1734–1755.). For qPCR, the Maxima SYBR Green qPCR Master Mix (2X) (ThermoFisher Scientific) was used with a Rotor-Gene 6000 (Corbett) apparatus. The *ydcD* primers were designed by using the PrimerQuest tool (IDT). Forward and reverse primer sequences (5’-3’) were GCACTGTGGAGTGGTTGATA and TGTAGCGCAGAACTCCATATTC. Two independent experiments were performed (ydcD duplicate 1 and 2). + and–RNase HI means that the genomic DNA was treated or not treated with RNase HI.
(PPTX)

S2 Fig MFA profile of genomic DNA from JB136 relative to JB137. The absolute coverage of genomic DNA from JB136 ((*ΔtopB ΔyncE topA20*::Tn*10 gyrB*(Ts) *IN(1*.*52–1*.*84)*) (Fig 3) relative to the absolute coverage from JB137 (*ΔtopB topA20*::Tn*10 gyrB*(Ts)) (Fig 2) is shown here.
(PPTX)

S3 Fig RLDR in strains JB260 and JB136. Flow cytometry to detect RLDR in JB260 (*ΔtopB ΔtusB topA20*::Tn*10 gyrB*(Ts)) and JB136 (*ΔtopB ΔyncE topA20*::Tn*10 gyrB*(Ts) *IN(1*.*52–1*.*84)*) cells grown at 30°C as described in Material and Methods. See the legend of Fig 6 for more details.
(PPTX)

S4 Fig *qseC/lepA* DNA ratio, and *parC/lepA* and *parE/lepA* RNA ratios in *topA* null and *topA topB* null strains carrying or not pET11-*parEC*. *qseC/lepA* ratio determined by qPCR of genomic DNA extracted from JB 206 (*topA20*::Tn*10 gyrB*(Ts)) and JB137 (*ΔtopB topA20*::Tn*10 gyrB*(Ts)) cells grown at 30°C to log phase as described in Material and Methods (A). *parC/lepA* and *parE/lepA* ratio determined by qRT-PCR of RNA extracted from RM443 (wild-type), RFM445 (*gyrB*(Ts)), JB137 (*ΔtopB topA20*::Tn*10 gyrB*(Ts)) and JB206 (*topA20*::Tn*10 gyrB*(Ts)) (B), and JB137 (*ΔtopB topA20*::Tn*10 gyrB*(Ts)) (value from (B)) and JB208 (JB137 pET11-*parEC*) (C). RNA extraction was performed has described using the RNAprotect Bacteria Reagent (Qiagen) and the RNeasy Mini kits (Qiagen) and the RNA preps were then treated with DNase (TURBO DNA-free kit from Invitrogen) (Brochu, J., Drolet, M. (2018) Topoisomerases I and III inhibit R-loop formation to prevent unregulated replication in the chromosomal Ter region of *Escherichia coli*. PLoS Genet. 2018 Sep 17;14(9):e1007668.). qRT-PCR using the QuantiNova SYBR Green RT-PCR kit (Qiagen) with the Rotor-Gene 6000 (Corbett) apparatus was performed as previously described (Brochu, J., Drolet, M. (2018) Topoisomerases I and III inhibit R-loop formation to prevent unregulated replication in the chromosomal Ter region of *Escherichia coli*. PLoS Genet. 2018 Sep 17;14(9):e1007668.). The *parC/lepA* and *parE/lepA* ratios were determined by using the 2^-Δct^ formula and standard deviations were calculated from these values. The primers were designed by using the PrimerQuest tool (IDT). Forward and reverse primer sequences (5’-3’) were GATGAACCACCTCTTCGCTAC and CAGCCATTCGGAGAGGATTT for *parC*, TCGGTAATTTCGCTGGTGATAC and CCCTGCATCGTTGGGATAAG for *parE* and GAGTACCGGGCAGACCTATAA and AGCCTACTTCGCCACATTTC for *lepA*. In (C), the topo IV activity overexpression levels for strain JB208 (JB137 pET11-*parEC*) were adjusted (delineated by the black horizontal lanes: the value of JB137 + the remaining value reduced by 5- to 10-fold) to take into account that the fusion protein produced from pET11-*parEC* is 5- to 10-fold less active than the normal protein (Lavasani LS, Hiasa H. (2001) A ParE-ParC fusion protein is a functional topoisomerase. Biochemistry Jul 24;40(29):8438–43.).
(PPTX)

S5 Fig A high level of topo IV overproduction due to a *parC parE* amplification allows a *topA topB* null derivative of MG1655 to grow well and to significantly reduce the Ter peak height, without inhibiting R-loop formation. (A) Cells of VS111 (MG1655 *ΔtopA*::*cam*), JB303 (VS111 *ΔtopB*::*kan*), JB350 (JB303 pSK760), JB352 (JB303 pSK762c) and RFM443 (wild-type) strains were grown overnight at 37°C on LB plates and diluted in fresh liquid LB medium for growth curve at 30°C. pSK760 but not pSK762c carries the wild-type *rnhA* gene to overproduce RNase HI. (B) Top: *qseC/lepA* ratio determined by qPCR of genomic DNA extracted from VS111 (MG1655 *ΔtopA*::*cam*) and JB303 (VS111 *ΔtopB*::*kan*) cells grown at 30°C to log phase as described in Materials and Methods. Bottom: *parC/lepA* and *parE/lepA* ratio determined by qRT-PCR of RNA extracted from VS111 (MG1655 *ΔtopA*::*cam*) and JB303 (VS111 *ΔtopB*::*kan*) cells. RNA extraction and qRT-PCR were performed as described in the legend of S4 Fig. (C) MFA by NGS of genomic DNA extracted from JB303 (VS111 *ΔtopB*::*kan*) cells grown at 30°C to log phase and treated (spc) or not treated (no spc) with spectinomycin for two hours. See the legend of Fig 2 for more detail. *amp* indicates the amplified DNA region carrying *parC* and *parE*. (d) Dot-blot with S9.6 antibodies of genomic DNA from JB303 (VS111 *ΔtopB*::*kan*), RFM443 (wild-type), and JB137 (*ΔtopB topA20*::Tn*10 gyrB*(Ts)) cells grown at 30°C. For JB303, various amounts of genomic DNA (as indicated) were spotted on the membrane. +RNase HI indicates that the genomic DNA was treated with RNase HI.
(PPTX)

S6 Fig High level of RLDR in the *topA topB* null derivative of MG1655. Flow cytometry to detect RLDR in JB303 (VS111 *ΔtopB*::*kan*), JB350 (JB303 pSK760), JB352 (JB303 pSK762c), and VS111 (MG1655 *ΔtopA*::*cam*) cells grown at 30°C as described in Materials and Methods. pSK760 but not pSK762c carries the wild-type *rnhA* gene to overproduce RNase HI. See the legend of Fig 6 for more details.
(PPTX)

S7 Fig Effects of a naturally occurring *gyrB* mutation (*gyrB225*) on the growth, DNA supercoiling and RLDR of *topA* null and *topA topB* null cells. (A) Cells of JB305 (DM800 (*Δ(topA cysB)204 gyrB225*) *ΔtopB*::*kan*), JB354 (JB305 pSK760), JB356 (JB305 pSK762c), and RFM443 (wild-type) were grown overnight at 37°C on LB plates and diluted in fresh liquid LB medium for growth curve at 30°C as described in Materials and Methods. (B) One-dimensional chloroquine gel electrophoresis (7.5 μg/ml of chloroquine) of pACYC184Δtet5’ extracted from DM800 (*Δ(topA cysB)204 gyrB225*)/pACYC184Δtet5’ and JB305 (DM800 *ΔtopB*::*kan*) /pACYC184Δtet5’ cells grown at 37°C to an OD_600_ of 0.4 (lanes 1 and 3), or 30 min after a transfer from 37 to 30°C (lanes 2 and 4) as described in Materials and Methods. Arrows indicate hyper-negatively supercoiled DNA. (C) Flow cytometry to detect RLDR in JB354 (JB305 pSK760), JB356 (JB305 pSK762c) and DM800 (*Δ(topA cysB)204 gyrB225*) cells grown at 30°C as described in Materials and Methods. See the legend of Fig 6 for more details. pSK760 but not pSK762c carries the wild-type *rnhA* gene to overproduce RNase HI.
(PDF)

S8 Fig Two-dimensional gel electrophoresis showing hypernegative supercoiling in strains JB206 and JB137. Two-dimensional chloroquine gel electrophoresis (7.5 and 30 μg/ml of chloroquine, respectively, in the first and second dimension) of pACYC184Δtet5’ extracted from JB206 (*topA20*::Tn*10 gyrB*(Ts))/pACYC184Δtet5’ and JB137 (*ΔtopB topA20*::Tn*10 gyrB*(Ts))/pACYC184Δtet5’ cells grown at 37°C to an OD_600_ of 0.4 and transferred to 30°C for 30 min as described in Materials and Methods. The gel was photographed by using the Blue light transilluminator (ThermoFisher scientific). Arrows indicate hyper-negatively supercoiled DNA.
(PDF)

S9 Fig The naturally occurring gyrB mutation (*gyrB225*) reduces the Ter peak height in *topA topB* null cells. MFA by NGS of genomic DNA extracted from JB305 (DM800 (*Δ(topA cysB)204 gyrB225*) *ΔtopB*::*kan*) cells grown at 30°C to log phase and treated (spc) or not treated (no spc) with spectinomycin for two hours. See the legend of Fig 2 for more detail.
(PPTX)

S10 Fig The rpoB*35 mutation inhibiting RNAP backtracking significantly corrects the filamentation and chromosome segregation defects of *topA topB* null cells. Cells of the JB639 ((*ΔtopB topA20*::Tn*10 gyrB*(Ts) rpo*35) strain were grown overnight at 37°C on LB plates and diluted in fresh liquid LB medium for growth at 30°C to an OD_600_ of 0.8 for microscopy, as described in Materials and Methods. (A) A representative merged image of phase contrast and fluorescence pictures of SYTO-40-stained cells. (B) Cells (total number in parentheses) were examined in merged images to calculate the percentage of cells in each category (numbers for JB137 were taken from Fig 4).
(PPTX)

S11 Fig Effect of deleting C-terminal portions of topo I on its ability to inhibit RLDR in *topA topB* null cells. Flow cytometry to detect RLDR in JB303 (VS111 (MG1655 *ΔtopA*::*cam*) *ΔtopB*::*kan)*, JB303/pJW312, JB303/pJW2277 and JB303 (pJW67) cells grown at 30°C as described in Materials and Methods. Results for JB303 were taken from S6 Fig. See the legend of Fig 6 for more details.
(PPTX)

S12 Fig The ability of topo I from *Mycobacterium sp*. to inhibit RLDR in *topA topB* null cells of *E*. *coli*. Flow cytometry to detect RLDR in JB137 (*ΔtopB topA20*::Tn*10 gyrB*(Ts)), JB137/p2OT-MOCR, JB137/p2OT-Msmtop, and JB137/p2OT-Mtbtop cells grown at 30°C as described in Materials and Methods. Results for JB137 were taken from Fig 6. See the legend of Fig 6 for more details.
(PDF)

S1 Table *Escherichia coli strains* and plasmids used in this work. The strains were constructed as described in Materials and Methods.
(DOCX)

S1 Numerical data qPCR and qRT-PCR numerical data. Ratios were determined by using the 2^-Δct^ formula as described in Material and Methods. Means and standard deviations from these values are also shown.
(XLSX)
