# Supplementary material for: Characterization of a pathway of genomic instability induced by R-loops and its regulation by topoisomerases in E. coli
Source: PLoS Genet. 2023 May 4;19(5):e1010754. doi: 10.1371/journal.pgen.1010754 (PMC10187895; doi:10.1371/journal.pgen.1010754)
Supplement: S1 Fig — Cells of MM62 (wild-type), MM84 (rnhA::cam) and VU425 (ΔtopB topA20::Tn10 gyrB(Ts)/pSK762c (Usongo V, Drolet M. (2014) Roles of type 1A topoisomerases in genome maintenance in Escherichia coli. PLoS Genet. 2014;10(8):e1004543.) strains were grown overnight at 37°C on LB plates and diluted in fresh liquid LB medium for growth at 30°C to an OD600 of 0.4. Genomic DNA was extracted by using the GenElute bacterial genomic DNA kit (Sigma Aldrich) as described by the manufacturer, except that 1 μl of RNase A instead of 20 was used. DRIP (DNA-RNA immunoprecipitation)-qPCR was performed exactly as described in Sanz and Chédin (Sanz LA, Chédin, F. (2019) High-resolution, strand-specific R-loop mapping via S9.6-based DNA-RNA immunoprecipitation and high-throughput sequencing. Nat Protoc. 2019 Jun;14(6):1734–1755.). For qPCR, the Maxima SYBR Green qPCR Master Mix (2X) (ThermoFisher Scientific) was used with a Rotor-Gene 6000 (Corbett) apparatus. The ydcD primers were designed by using the PrimerQuest tool (IDT). Forward and reverse primer sequences (5’-3’) were GCACTGTGGAGTGGTTGATA and TGTAGCGCAGAACTCCATATTC. Two independent experiments were performed (ydcD duplicate 1 and 2). + and–RNase HI means that the genomic DNA was treated or not treated with RNase HI. (PPTX) [file pgen.1010754.s001.pptx]

## Slide 1
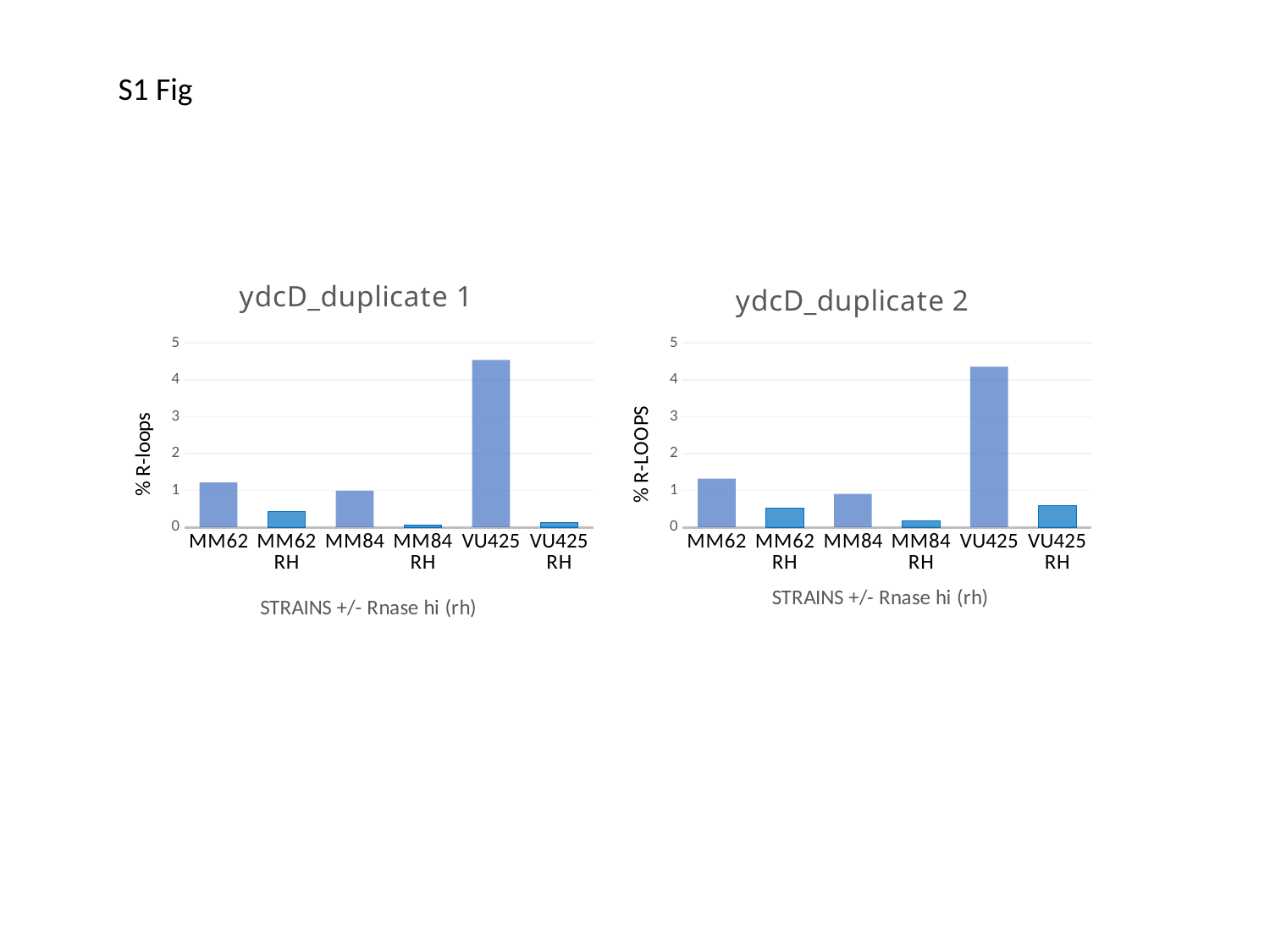

S1 Fig
### Chart: ydcD_duplicate 1
| Category | sDRIP |
|---|---|
| MM62 | 1.2158186842653531 |
| MM62 RH | 0.4328467087846635 |
| MM84 | 0.9944206046936472 |
| MM84 RH | 0.051901789613144914 |
| VU425 | 4.537595776585803 |
| VU425 RH | 0.12430257558670608 |
### Chart: ydcD_duplicate 2
| Category | sDRIP |
|---|---|
| MM62 | 1.3212725507017258 |
| MM62 RH | 0.5147443857922338 |
| MM84 | 0.9087328233251954 |
| MM84 RH | 0.16862941195381698 |
| VU425 | 4.352752816480636 |
| VU425 RH | 0.59539874877746 |
