# Supplementary figures and images for: Characterization of a pathway of genomic instability induced by R-loops and its regulation by topoisomerases in E. coli
Source: PLoS Genet. 2023 May 4;19(5):e1010754. doi: 10.1371/journal.pgen.1010754 (PMC10187895; doi:10.1371/journal.pgen.1010754)

## Slide 1
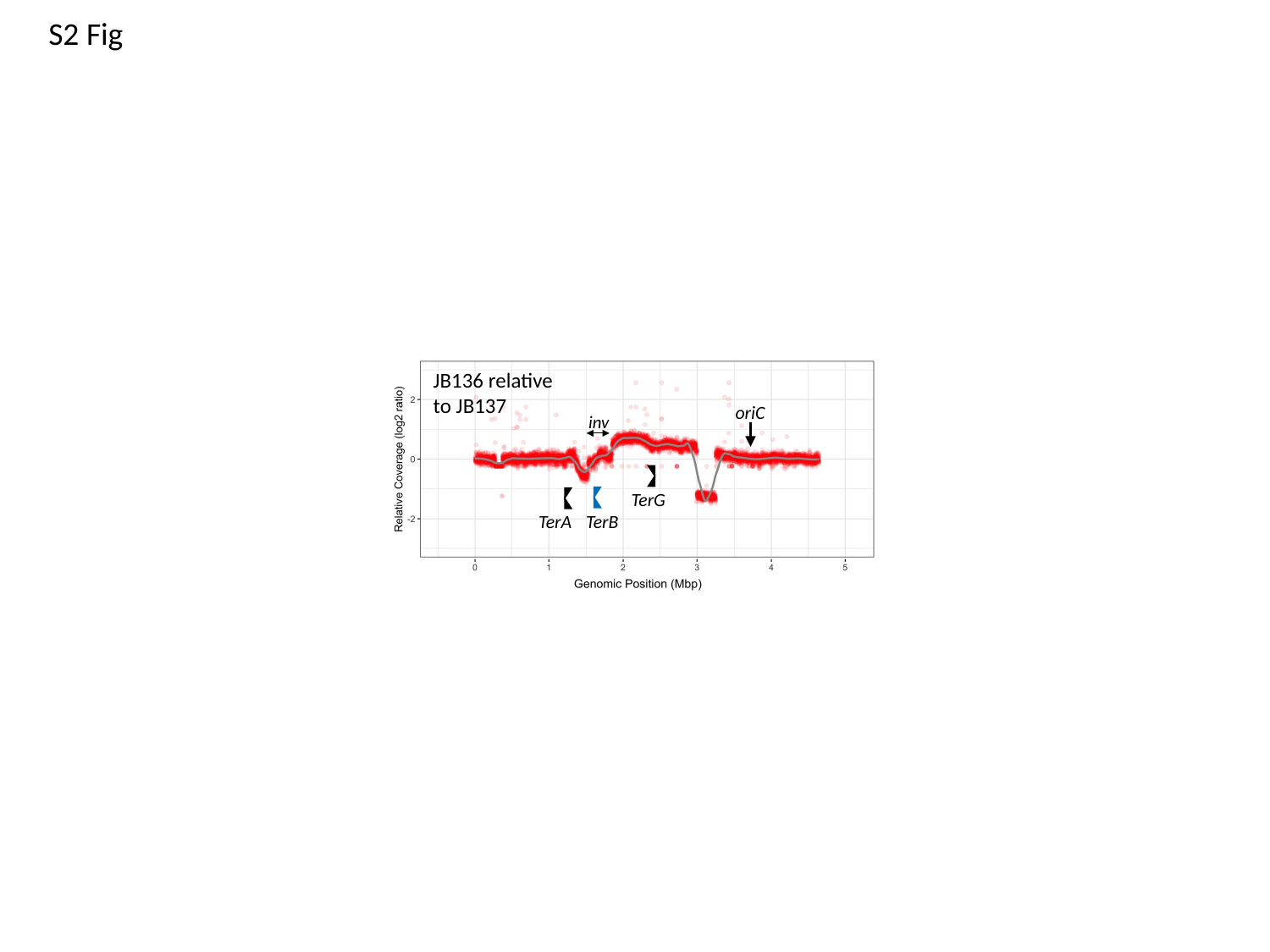

S2 Fig
JB136 relative to JB137
oriC
inv
TerG
TerA
TerB

Supplement: S2 Fig — The absolute coverage of genomic DNA from JB136 ((ΔtopB ΔyncE topA20::Tn10 gyrB(Ts) IN(1.52–1.84)) (Fig 3) relative to the absolute coverage from JB137 (ΔtopB topA20::Tn10 gyrB(Ts)) (Fig 2) is shown here. (PPTX) [file pgen.1010754.s002.pptx]

## Slide 1
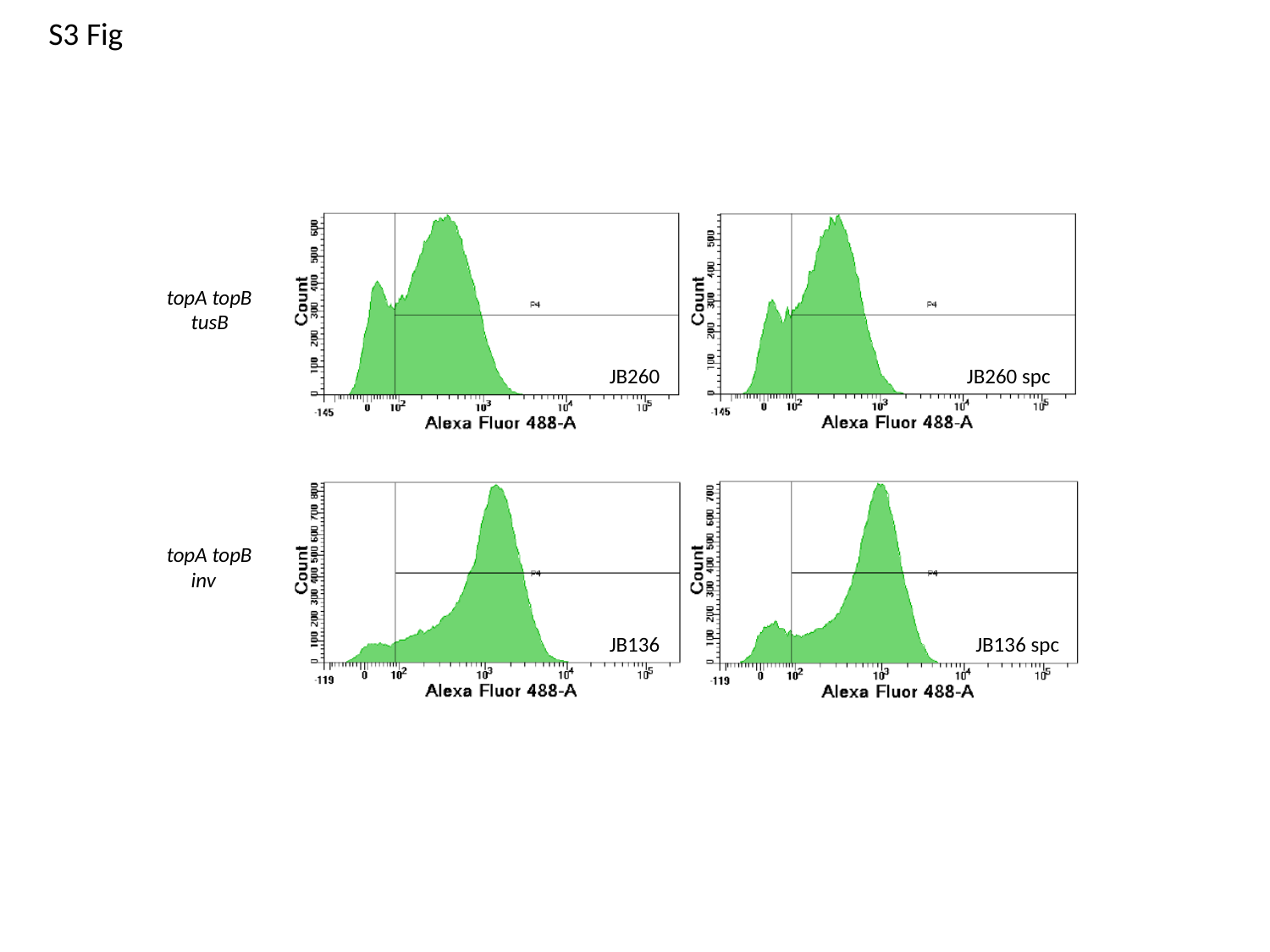

S3 Fig
topA topB
 tusB
JB260
JB260 spc
topA topB
 inv
JB136 spc
JB136

Supplement: S3 Fig — Flow cytometry to detect RLDR in JB260 (ΔtopB ΔtusB topA20::Tn10 gyrB(Ts)) and JB136 (ΔtopB ΔyncE topA20::Tn10 gyrB(Ts) IN(1.52–1.84)) cells grown at 30°C as described in Material and Methods. See the legend of Fig 6 for more details. (PPTX) [file pgen.1010754.s003.pptx]

S7 Fig

**A**

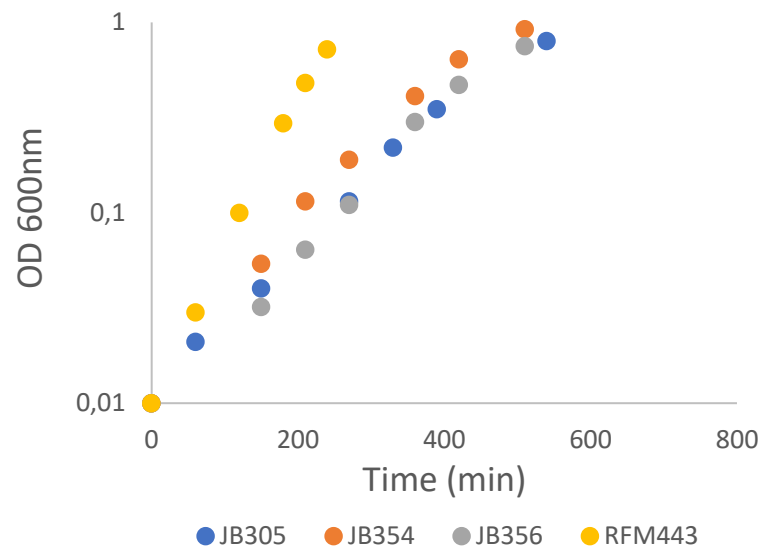

**B**

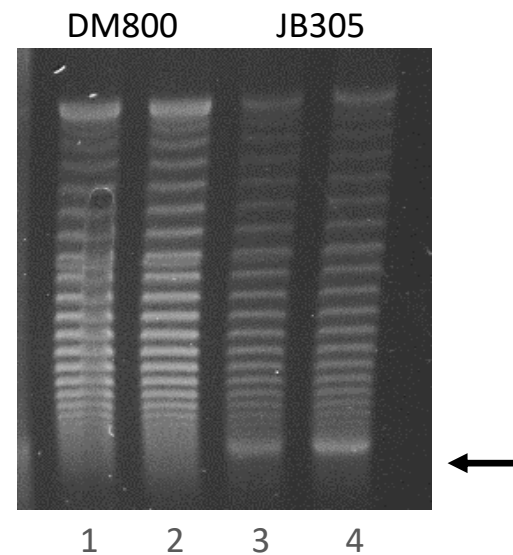

**C**

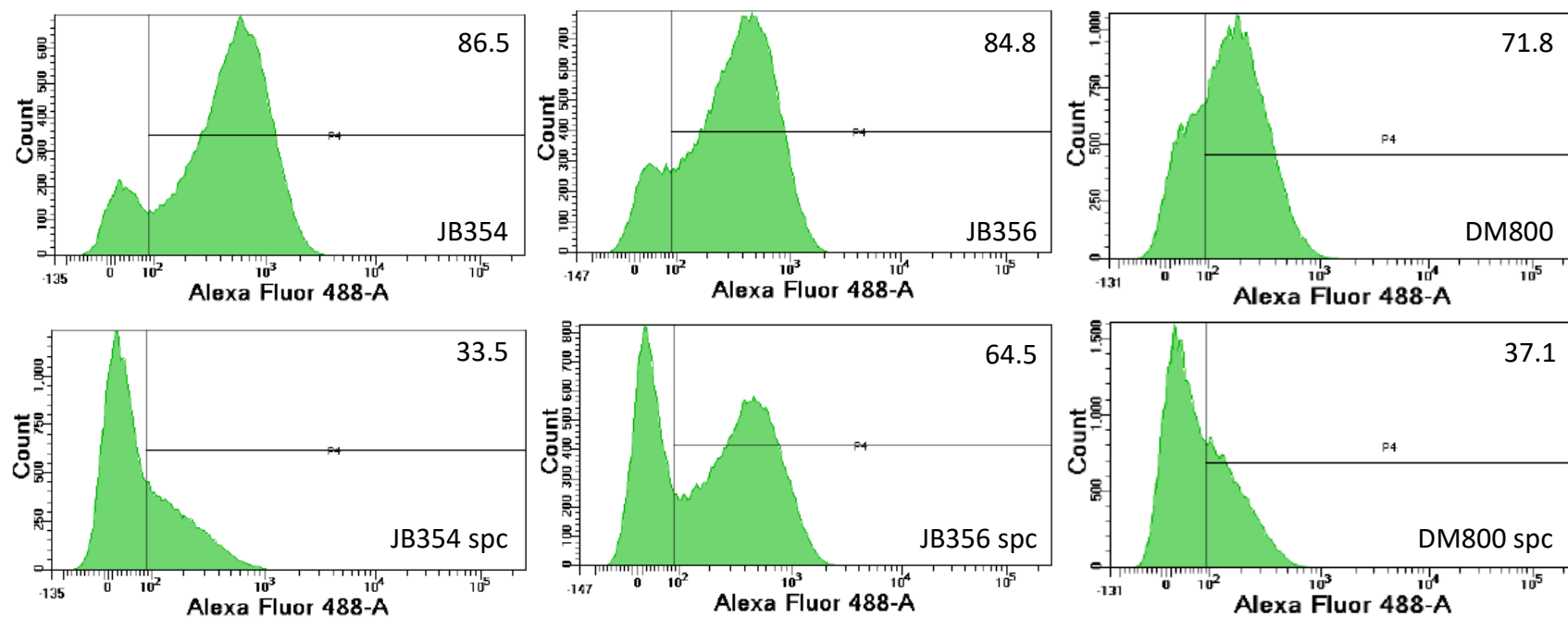

Supplement: S7 Fig — (A) Cells of JB305 (DM800 (Δ(topA cysB)204 gyrB225) ΔtopB::kan), JB354 (JB305 pSK760), JB356 (JB305 pSK762c), and RFM443 (wild-type) were grown overnight at 37°C on LB plates and diluted in fresh liquid LB medium for growth curve at 30°C as described in Materials and Methods. (B) One-dimensional chloroquine gel electrophoresis (7.5 μg/ml of chloroquine) of pACYC184Δtet5’ extracted from DM800 (Δ(topA cysB)204 gyrB225)/pACYC184Δtet5’ and JB305 (DM800 ΔtopB::kan) /pACYC184Δtet5’ cells grown at 37°C to an OD600 of 0.4 (lanes 1 and 3), or 30 min after a transfer from 37 to 30°C (lanes 2 and 4) as described in Materials and Methods. Arrows indicate hyper-negatively supercoiled DNA. (C) Flow cytometry to detect RLDR in JB354 (JB305 pSK760), JB356 (JB305 pSK762c) and DM800 (Δ(topA cysB)204 gyrB225) cells grown at 30°C as described in Materials and Methods. See the legend of Fig 6 for more details. pSK760 but not pSK762c carries the wild-type rnhA gene to overproduce RNase HI. (PDF) [file pgen.1010754.s007.pdf]

S8 Fig

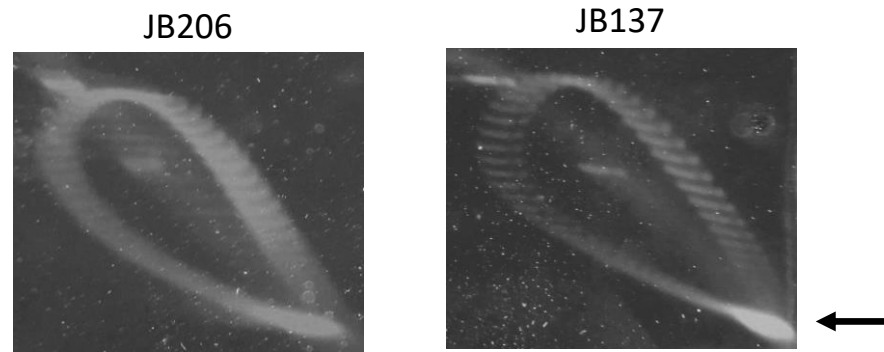

Supplement: S8 Fig — Two-dimensional chloroquine gel electrophoresis (7.5 and 30 μg/ml of chloroquine, respectively, in the first and second dimension) of pACYC184Δtet5’ extracted from JB206 (topA20::Tn10 gyrB(Ts))/pACYC184Δtet5’ and JB137 (ΔtopB topA20::Tn10 gyrB(Ts))/pACYC184Δtet5’ cells grown at 37°C to an OD600 of 0.4 and transferred to 30°C for 30 min as described in Materials and Methods. The gel was photographed by using the Blue light transilluminator (ThermoFisher scientific). Arrows indicate hyper-negatively supercoiled DNA. (PDF) [file pgen.1010754.s008.pdf]

## Slide 1
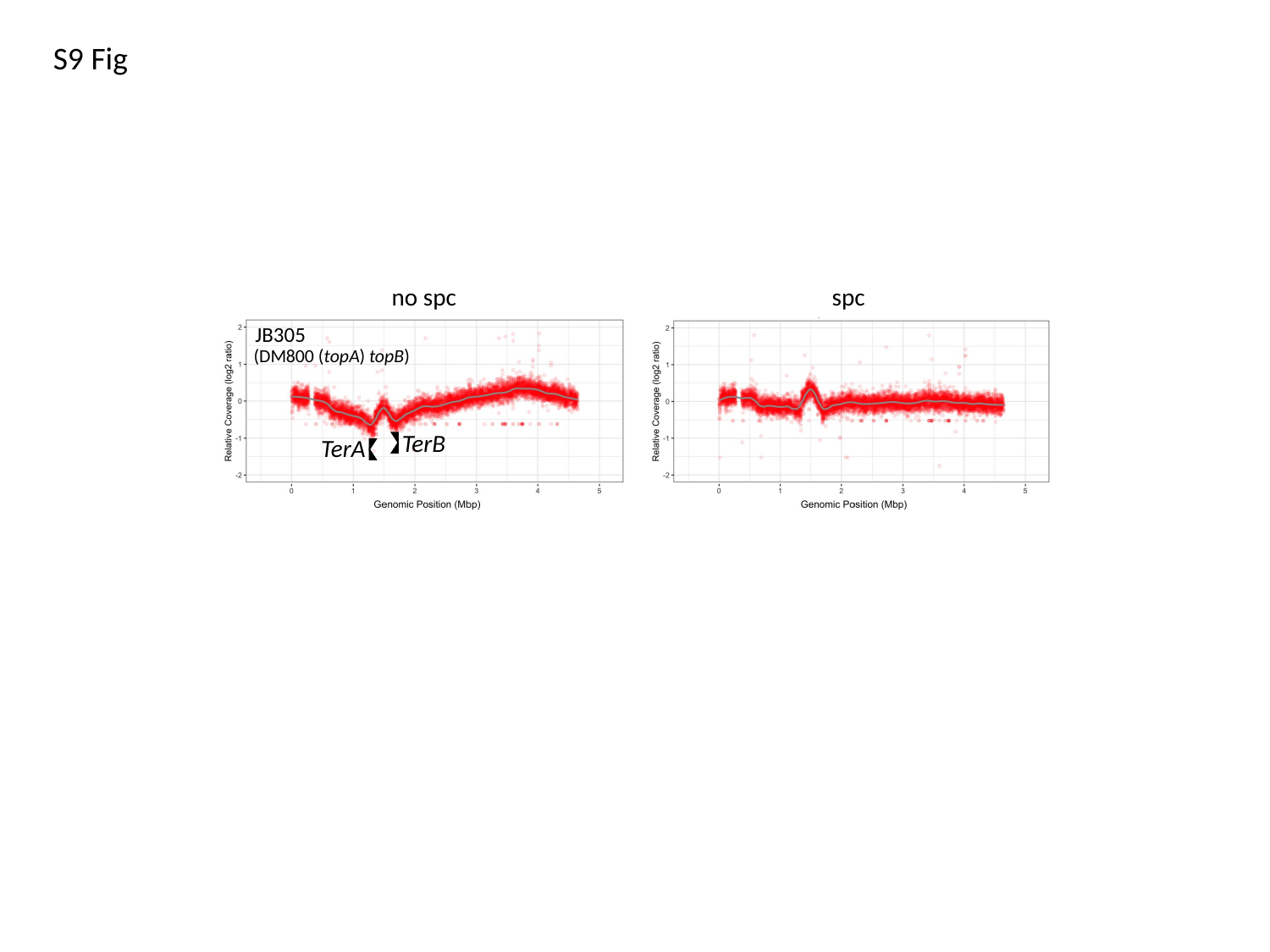

S9 Fig
no spc
spc
JB305
(DM800 (topA) topB)
TerB
TerA

Supplement: S9 Fig — MFA by NGS of genomic DNA extracted from JB305 (DM800 (Δ(topA cysB)204 gyrB225) ΔtopB::kan) cells grown at 30°C to log phase and treated (spc) or not treated (no spc) with spectinomycin for two hours. See the legend of Fig 2 for more detail. (PPTX) [file pgen.1010754.s009.pptx]

## Slide 1
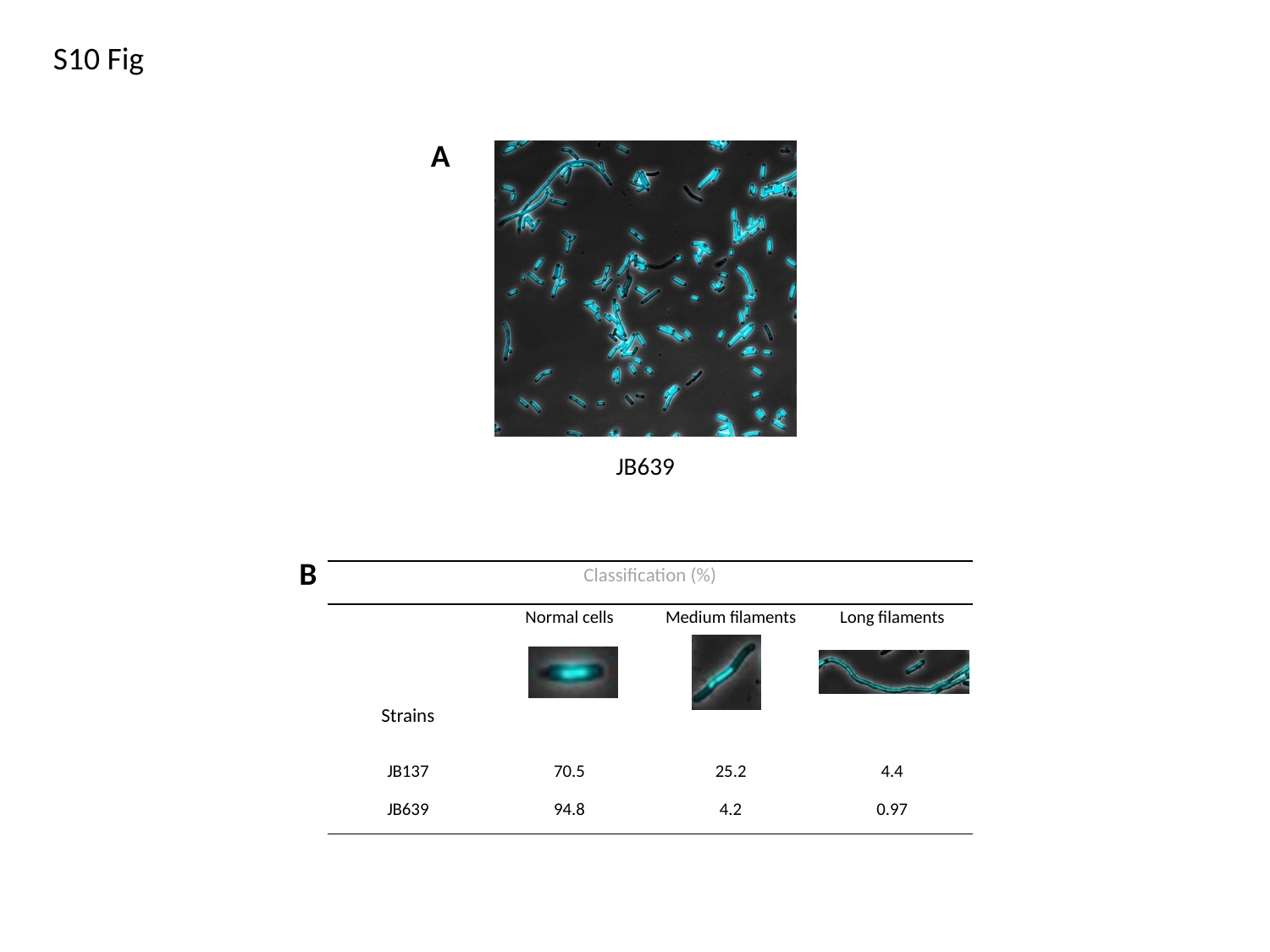

S10 Fig
A
JB639
B
| Classification (%) | | | |
| --- | --- | --- | --- |
| | Normal cells | Medium filaments | Long filaments |
| Strains | | | |
| JB137 | 70.5 | 25.2 | 4.4 |
| JB639 | 94.8 | 4.2 | 0.97 |

Supplement: S10 Fig — Cells of the JB639 ((ΔtopB topA20::Tn10 gyrB(Ts) rpo*35) strain were grown overnight at 37°C on LB plates and diluted in fresh liquid LB medium for growth at 30°C to an OD600 of 0.8 for microscopy, as described in Materials and Methods. (A) A representative merged image of phase contrast and fluorescence pictures of SYTO-40-stained cells. (B) Cells (total number in parentheses) were examined in merged images to calculate the percentage of cells in each category (numbers for JB137 were taken from Fig 4). (PPTX) [file pgen.1010754.s010.pptx]

## Slide 1
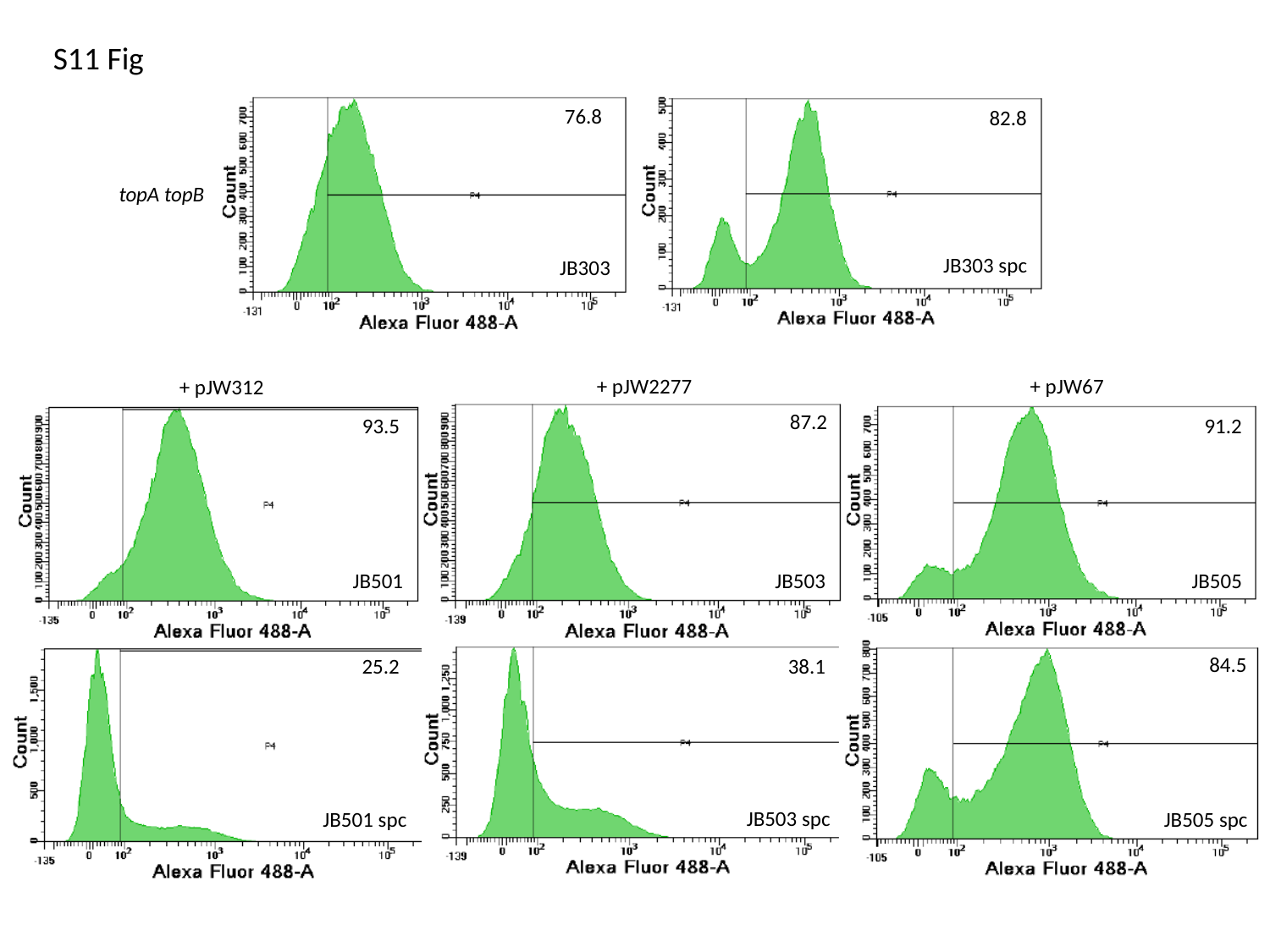

S11 Fig
76.8
82.8
topA topB
JB303 spc
JB303
+ pJW2277
+ pJW67
+ pJW312
87.2
93.5
91.2
JB503
JB501
JB505
84.5
38.1
25.2
JB503 spc
JB505 spc
JB501 spc

Supplement: S11 Fig — Flow cytometry to detect RLDR in JB303 (VS111 (MG1655 ΔtopA::cam) ΔtopB::kan), JB303/pJW312, JB303/pJW2277 and JB303 (pJW67) cells grown at 30°C as described in Materials and Methods. Results for JB303 were taken from S6 Fig. See the legend of Fig 6 for more details. (PPTX) [file pgen.1010754.s011.pptx]

# S12 Fig

*topA topB*

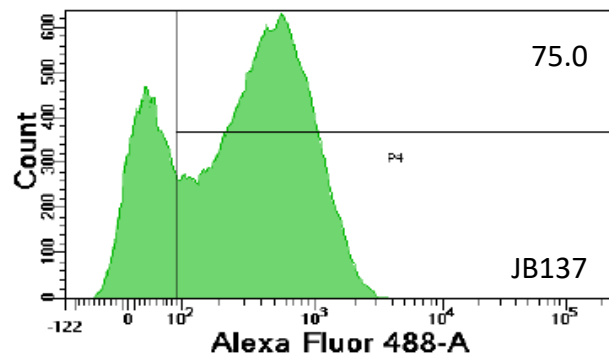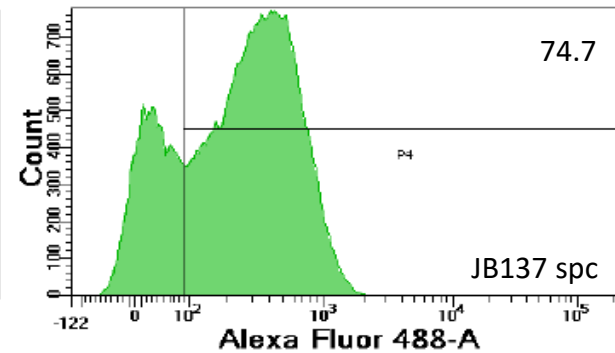

+ p2OT-MOCR

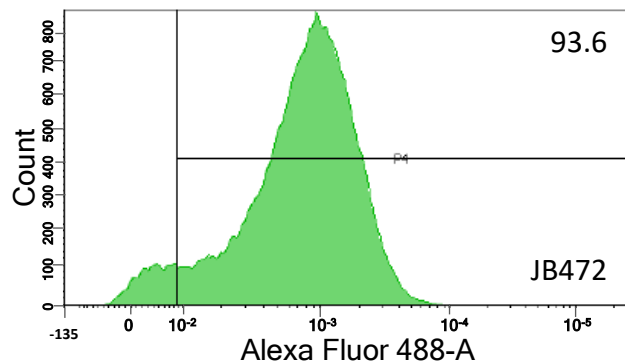

+ p2OT-Msmtop

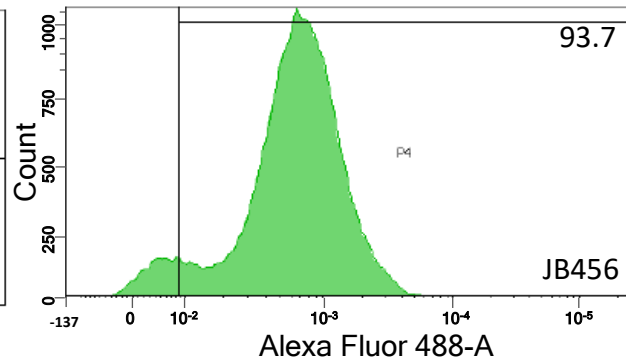

+ p2OT-Mtbttop

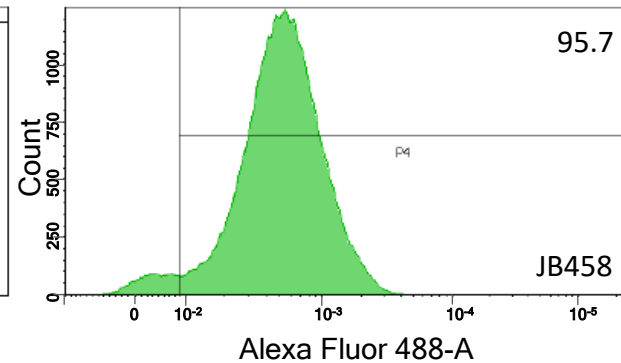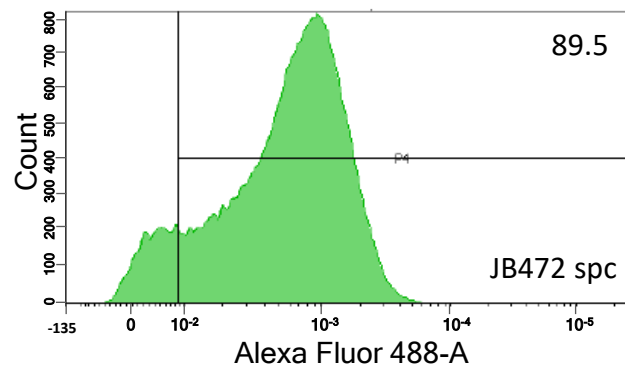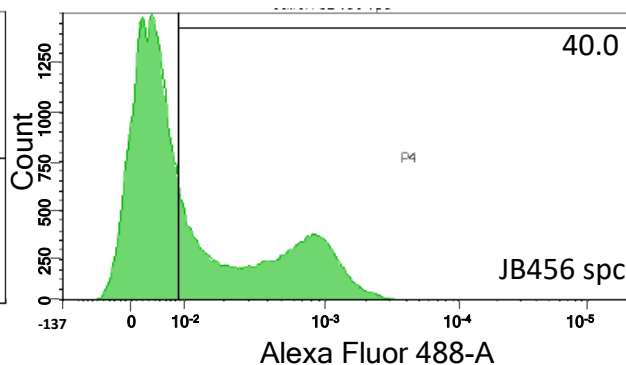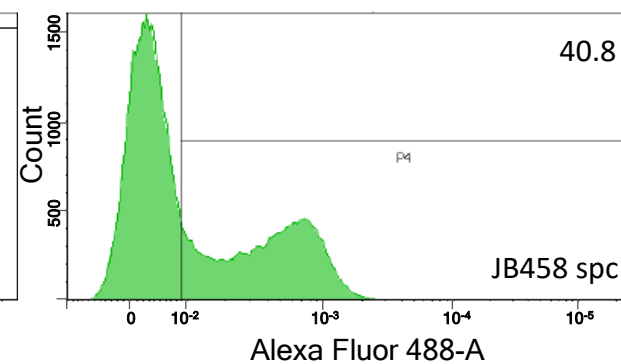

Supplement: S12 Fig — Flow cytometry to detect RLDR in JB137 (ΔtopB topA20::Tn10 gyrB(Ts)), JB137/p2OT-MOCR, JB137/p2OT-Msmtop, and JB137/p2OT-Mtbtop cells grown at 30°C as described in Materials and Methods. Results for JB137 were taken from Fig 6. See the legend of Fig 6 for more details. (PDF) [file pgen.1010754.s012.pdf]
