# Supplementary material for: Characterization of a pathway of genomic instability induced by R-loops and its regulation by topoisomerases in E. coli
Source: PLoS Genet. 2023 May 4;19(5):e1010754. doi: 10.1371/journal.pgen.1010754 (PMC10187895; doi:10.1371/journal.pgen.1010754)
Supplement: S4 Fig — qseC/lepA ratio determined by qPCR of genomic DNA extracted from JB 206 (topA20::Tn10 gyrB(Ts)) and JB137 (ΔtopB topA20::Tn10 gyrB(Ts)) cells grown at 30°C to log phase as described in Material and Methods (A). parC/lepA and parE/lepA ratio determined by qRT-PCR of RNA extracted from RM443 (wild-type), RFM445 (gyrB(Ts)), JB137 (ΔtopB topA20::Tn10 gyrB(Ts)) and JB206 (topA20::Tn10 gyrB(Ts)) (B), and JB137 (ΔtopB topA20::Tn10 gyrB(Ts)) (value from (B)) and JB208 (JB137 pET11-parEC) (C). RNA extraction was performed has described using the RNAprotect Bacteria Reagent (Qiagen) and the RNeasy Mini kits (Qiagen) and the RNA preps were then treated with DNase (TURBO DNA-free kit from Invitrogen) (Brochu, J., Drolet, M. (2018) Topoisomerases I and III inhibit R-loop formation to prevent unregulated replication in the chromosomal Ter region of Escherichia coli. PLoS Genet. 2018 Sep 17;14(9):e1007668.). qRT-PCR using the QuantiNova SYBR Green RT-PCR kit (Qiagen) with the Rotor-Gene 6000 (Corbett) apparatus was performed as previously described (Brochu, J., Drolet, M. (2018) Topoisomerases I and III inhibit R-loop formation to prevent unregulated replication in the chromosomal Ter region of Escherichia coli. PLoS Genet. 2018 Sep 17;14(9):e1007668.). The parC/lepA and parE/lepA ratios were determined by using the 2-Δct formula and standard deviations were calculated from these values. The primers were designed by using the PrimerQuest tool (IDT). Forward and reverse primer sequences (5’-3’) were GATGAACCACCTCTTCGCTAC and CAGCCATTCGGAGAGGATTT for parC, TCGGTAATTTCGCTGGTGATAC and CCCTGCATCGTTGGGATAAG for parE and GAGTACCGGGCAGACCTATAA and AGCCTACTTCGCCACATTTC for lepA. In (C), the topo IV activity overexpression levels for strain JB208 (JB137 pET11-parEC) were adjusted (delineated by the black horizontal lanes: the value of JB137 + the remaining value reduced by 5- to 10-fold) to take into account that the fusion protein produced from pET11-parEC is 5- to 10-fold less active than [file pgen.1010754.s004.pptx]

## Slide 1
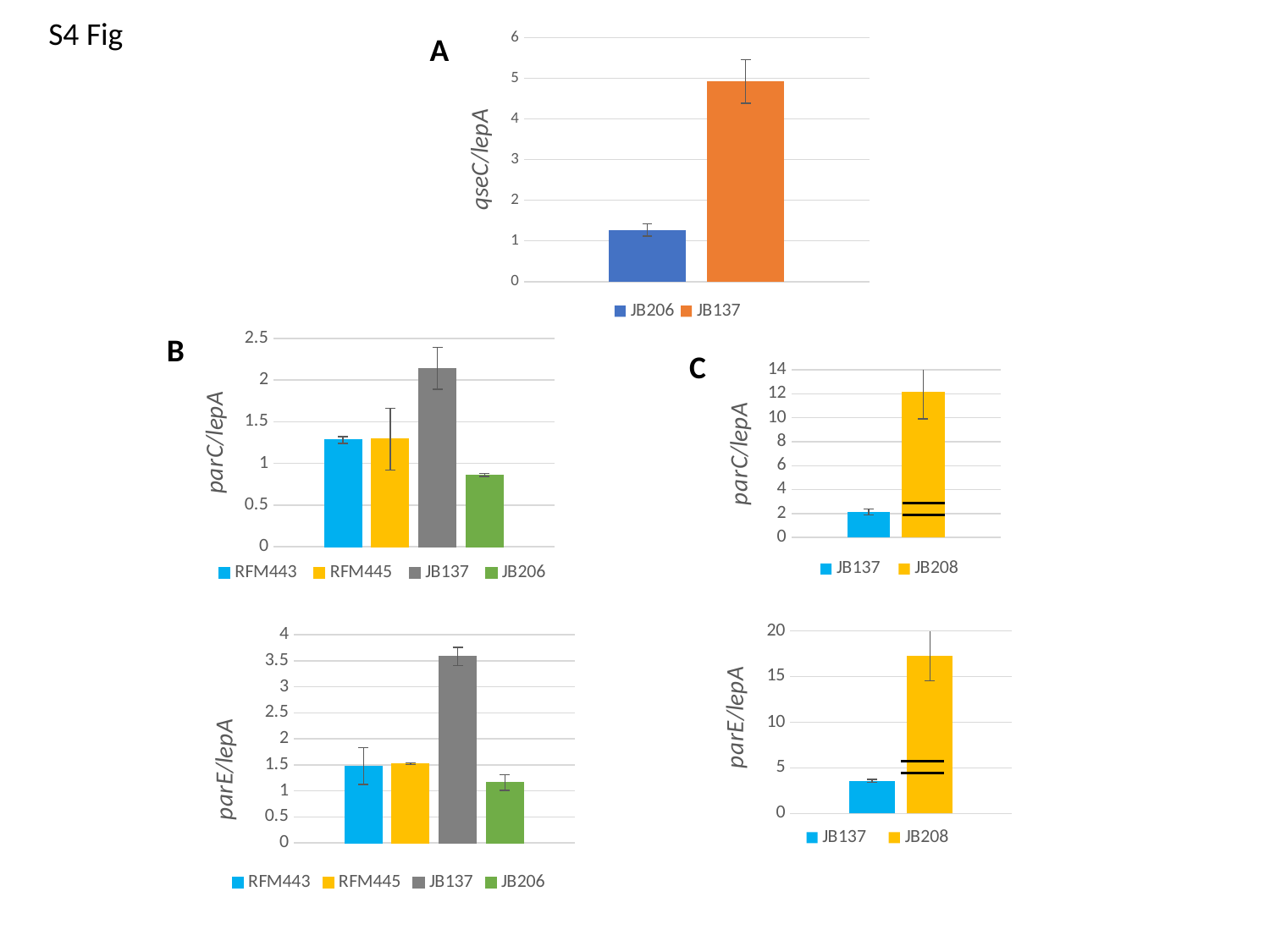

S4 Fig
A
### Chart
| Category | JB206 | JB137 |
|---|---|---|
### Chart
| Category | RFM443 | RFM445 | JB137 | JB206 |
|---|---|---|---|---|B
C
### Chart
| Category | JB137 | JB208 |
|---|---|---|
### Chart
| Category | JB137 | JB208 |
|---|---|---|
### Chart
| Category | RFM443 | RFM445 | JB137 | JB206 |
|---|---|---|---|---|
