# Supplementary material for: Characterization of a pathway of genomic instability induced by R-loops and its regulation by topoisomerases in E. coli
Source: PLoS Genet. 2023 May 4;19(5):e1010754. doi: 10.1371/journal.pgen.1010754 (PMC10187895; doi:10.1371/journal.pgen.1010754)
Supplement: S5 Fig — (A) Cells of VS111 (MG1655 ΔtopA::cam), JB303 (VS111 ΔtopB::kan), JB350 (JB303 pSK760), JB352 (JB303 pSK762c) and RFM443 (wild-type) strains were grown overnight at 37°C on LB plates and diluted in fresh liquid LB medium for growth curve at 30°C. pSK760 but not pSK762c carries the wild-type rnhA gene to overproduce RNase HI. (B) Top: qseC/lepA ratio determined by qPCR of genomic DNA extracted from VS111 (MG1655 ΔtopA::cam) and JB303 (VS111 ΔtopB::kan) cells grown at 30°C to log phase as described in Materials and Methods. Bottom: parC/lepA and parE/lepA ratio determined by qRT-PCR of RNA extracted from VS111 (MG1655 ΔtopA::cam) and JB303 (VS111 ΔtopB::kan) cells. RNA extraction and qRT-PCR were performed as described in the legend of S4 Fig. (C) MFA by NGS of genomic DNA extracted from JB303 (VS111 ΔtopB::kan) cells grown at 30°C to log phase and treated (spc) or not treated (no spc) with spectinomycin for two hours. See the legend of Fig 2 for more detail. amp indicates the amplified DNA region carrying parC and parE. (d) Dot-blot with S9.6 antibodies of genomic DNA from JB303 (VS111 ΔtopB::kan), RFM443 (wild-type), and JB137 (ΔtopB topA20::Tn10 gyrB(Ts)) cells grown at 30°C. For JB303, various amounts of genomic DNA (as indicated) were spotted on the membrane. +RNase HI indicates that the genomic DNA was treated with RNase HI. (PPTX) [file pgen.1010754.s005.pptx]

## Slide 1
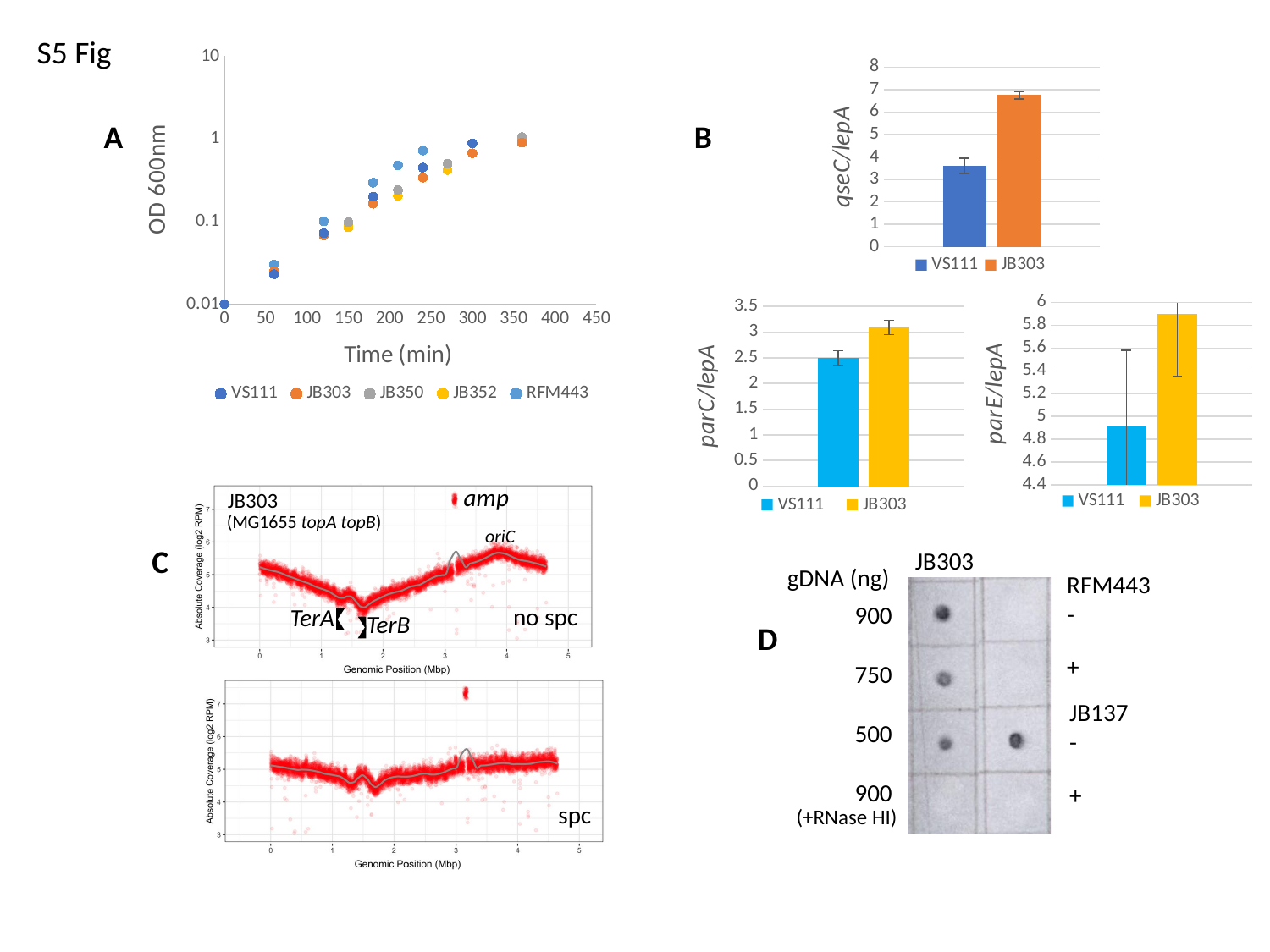

S5 Fig
### Chart
| Category | VS111 | JB303 | JB350 | JB352 | RFM443 |
|---|---|---|---|---|---|
### Chart
| Category | VS111 | JB303 |
|---|---|---|A
B
### Chart
| Category | VS111 | JB303 |
|---|---|---|
### Chart
| Category | VS111 | JB303 |
|---|---|---|amp
JB303
(MG1655 topA topB)
oriC
C
JB303
gDNA (ng)
RFM443
-
+
900
750
500
900
D
JB137
-
+
(+RNase HI)
no spc
TerA
TerB
spc
