# Supplementary material for: Characterization of a pathway of genomic instability induced by R-loops and its regulation by topoisomerases in E. coli
Source: PLoS Genet. 2023 May 4;19(5):e1010754. doi: 10.1371/journal.pgen.1010754 (PMC10187895; doi:10.1371/journal.pgen.1010754)
Supplement: S6 Fig — Flow cytometry to detect RLDR in JB303 (VS111 ΔtopB::kan), JB350 (JB303 pSK760), JB352 (JB303 pSK762c), and VS111 (MG1655 ΔtopA::cam) cells grown at 30°C as described in Materials and Methods. pSK760 but not pSK762c carries the wild-type rnhA gene to overproduce RNase HI. See the legend of Fig 6 for more details. (PPTX) [file pgen.1010754.s006.pptx]

## Slide 1
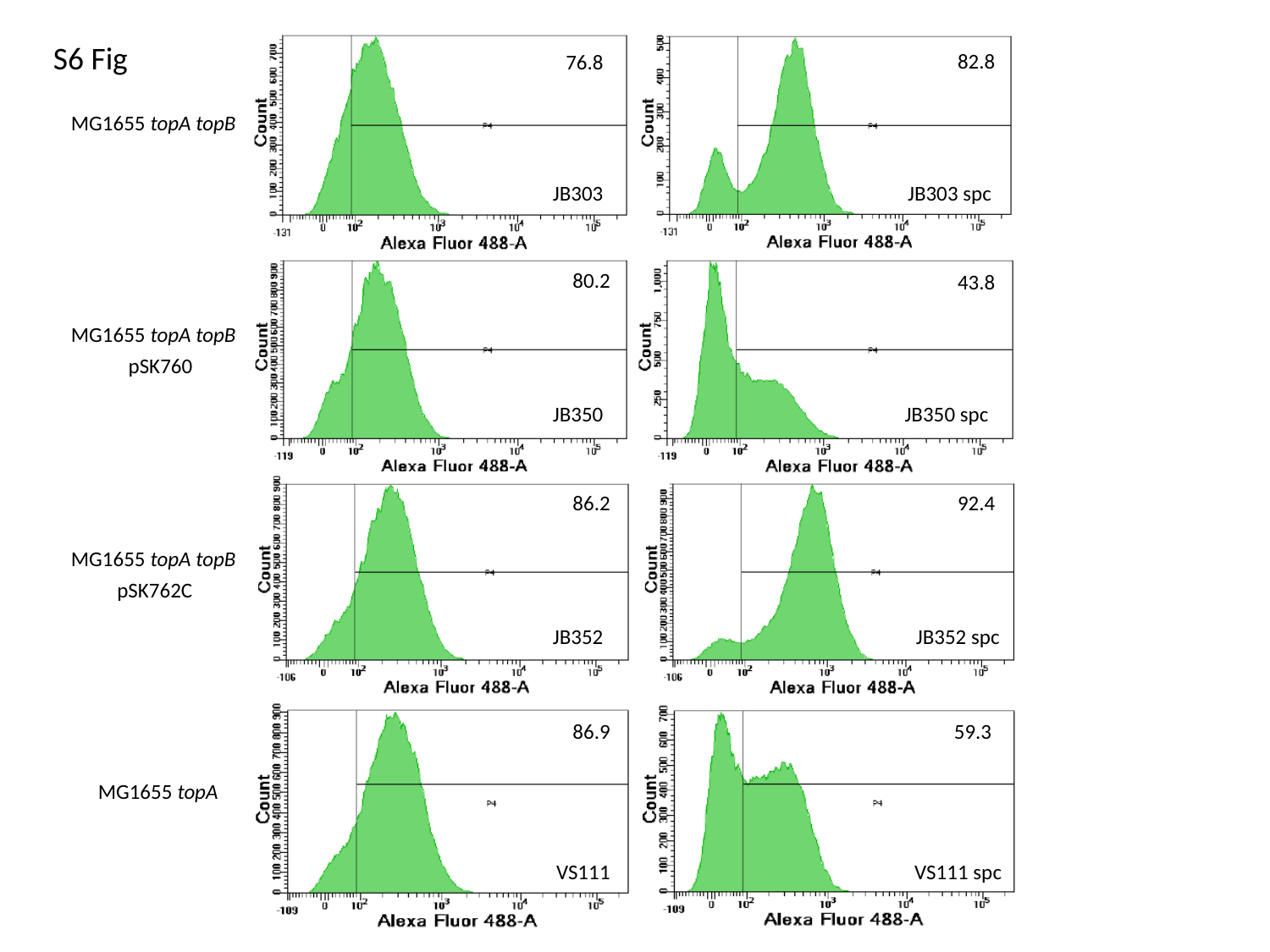

S6 Fig
82.8
76.8
MG1655 topA topB
JB303 spc
JB303
80.2
43.8
MG1655 topA topB
pSK760
JB350 spc
JB350
92.4
86.2
MG1655 topA topB
pSK762C
JB352
JB352 spc
86.9
59.3
MG1655 topA
VS111
VS111 spc
