# Supplementary material for: Characterization of a pathway of genomic instability induced by R-loops and its regulation by topoisomerases in E. coli
Source: PLoS Genet. 2023 May 4;19(5):e1010754. doi: 10.1371/journal.pgen.1010754 (PMC10187895; doi:10.1371/journal.pgen.1010754)
Supplement: S1 Table — The strains were constructed as described in Materials and Methods. (DOCX) [file pgen.1010754.s013.docx]

**S1 Table**. *Escherichia coli* strains and plasmids used.

| Name | Genotype or Relevant Genotype | Reference or Source |
| --- | --- | --- |
| CT77  CT170  EV1  EV3  DM800  JB55  JB120  JB121  JB134  JB136  JB137  JB177  JB185  JB187  JB194  JB206  JB208  JB260  JB303  JB305  JB335  JB350  JB352  JB354  JB356  JB393  JB395  JB456  JB458  JB472  JB475  JB501  JB503  JB505  JB511  JB512  JB568  JB607  JB626  JB631  JB639  JB656  JB657  JW1446-1  JW1447-1  JW1452-1  JW1602-3  JW1752-1  MD897  MM84  RFM443  RFM445  RFM480  VS111  VU409  VU410  VU411  pACYC184∆*tet*5’  pSK760  pSK762c  pET11-*parEC* | RFM443 *ΔtopB::kan*  RFM475 *ΔtopB::kan*  VU409 pSK760  VU409 pSK762c  *∆(topA cysB)204 gyrB225 acrA13*  VU409 *ΔyncD::kan*  VU410 *ΔyncE::kan*  VU410 *ΔyncE*  JB55 *topA20::*Tn*10*  JB121 *topA20::*Tn*10 IN(1.52-1.84)*  VU411 *topA20::*Tn*10*  VU409 *ΔyncE::kan*  VU409 *ΔyncE*  VU409 *ΔtusB ::kan*  VU409 *ΔtusB*  RFM445 *topA20::*Tn*10*  JB137 pET11-*parEC*  JB194 *topA20::*Tn*10*  VS111 *ΔtopB::kan*  DM800 *ΔtopB::kan*  JB185 *topA20::*Tn*10*  JB303 pSK760  JB303 pSK762c  JB305 pSK760  JB305 pSK762c  JB137 pSK760  JB137 pSK762c  JB137 p2OT-Msmtop  JB137 p2OT-Mtbtop  JB137 p2OT-MOCR  MG1655 *argF* *ΔlacZ* *rpo*35* *btuB*::Tn*10*  JB303 pJW312  JB303 pJW2277  JB303 pJW67  EV1 *topA20::*Tn*10*  EV3 *topA20::*Tn*10*  RFM445 *rpo*35* *btuB*::Tn*10*  JB568 *ΔtopB*  VU409 *ΔydcD::kan*  JB626 *topA20::*Tn*10*  JB607 *ΔtopA::cam*  JB639 pSK760  JB639 pSK762c  *ΔyncD743::kan*  Δ*yncE744::kan*  *ΔydcD750::kan*  Δ*tus758::kan*  *ΔtopB761::kan*  DM4100 Δ*topB::kan*  RFM443 *rnhA::cam*  *Δ(codB-lacI)3 rpsL200 galK2*(Oc) *IN(rrnD-rrnE)1 rph-1*  *Δ(codB-lacI)3 rpsL200 galK2*(Oc) *IN(rrnD-rrnE)1 rph-1 gyrB221* (Cou^r^) *gyrB203*(Ts)  *Δ(codB-lacI)3, rpsL200, galK2*(Oc), *IN(rrnD-rrnE)1, rph-1 gyrB221* (Cou^r^) *gyrB203*(Ts) *topA20*::Tn*10*  MG1655 *ΔtopA::cam*  RFM445 *ΔtopB*  RFM445 *ΔtopB*  RFM445 *ΔtopB*  deletion of the 5’ portion of the *tetA* gene that inactivates its translation.  *rnhA* gene with its own promoter  like pSK760 but *rnhA* is mutated and  inactive  production of an active ParEC fusion protein | RFM443 x P1(MD897)  (1)  This work  This work  (2)  VU409 x P1(JW1446-1)  VU410 x P1(JW1447-1)  JB120, *kan* removed by pCP20  JB55 x P1(RFM480)  JB121 x P1(RFM480)  VU411 x P1(RFM480)  VU409 x P1(JW1447-1)  JB177, *kan* removed by pCP20  VU409 x P1(JW1602-3)  JB187, *kan* removed by pCP20  RFM445 x P1(RFM480)  This work  JB194 x P1(RFM480)  VS111 x P1(JW1752-1)  DM800 x P1(MD897)  JB185 x P1(RFM480)  This work  This work  This work  This work  This work  This work  This work  This work  This work  (3)  This work  This work  This work  EV1 x P1(RFM480)  EV3 x P1(RFM480)  RFM445 x P1(JB475)^a^  JB568 x P1(JW1752-1) *kan* removed by pCP20  VU409 x P1(JW1452-1)  JB626 x P1(RFM480)  JB607 x P1(VS111)  This work  This work  (4)  (4)  (4)  (4)  (4)  Lab collection  (5)  (6)  (6)  (6)  (7)  (8)^b^  (8)^b^  (8)^b^  (9)  (6)  (6)  (10) |

pJW312 *E. coli topA* gene with its own promoter (11)

pJW67 67 kDa N-terminal portion of *E. coli* (11)

topo I.

pJW2277 85 kDa N-terminal portion of *E. coli* (12)

topo I (lack the 14-kDa C-terminal

fragment)

p2OT-MOCR control, empty vector (https://www.addgene.org/29710)

p2OT-Msmtop expressing *Mycobacterium smegmatis* (13)

topo I.

p2OT-Mtbtop expressing *Mycobacterium tuberculosis* (13)

topo I.

^a^Selected on LB plates with rifampicin (15 µg/ml) and the presence of *rpo*35* was confirmed by sequencing.

^b^Three clones of the transduction experiment to introduce a *topB* deletion in RFM445.

**References**

1. Usongo V, Tanguay C, Nolent F, Egbe Bessong J, Drolet M (2013) Interplay between type 1A topoisomerases and gyrase in chromosome segregation in *Escherichia coli*. J Bacteriol 195:1758-1768.
2. Sternglanz R, DiNardo S, Voelkel KA, Nishimura Y, Hirota Y, Becherer K, Zumstein L, Wang JC (1981) Mutations in the gene coding for *Escherichia coli* DNA topoisomerase I affect transcription and transposition. Proc Natl Acad Sci U S A. 78:2747-2751.
3. Leela JK, Raghunathan N, Gowrishankar J (2021). Topoisomerase I Essentiality, DnaA-Independent Chromosomal Replication, and Transcription-Replication Conflict in *Escherichia coli*. J Bacteriol. Aug 9;203(17):e0019521. doi: 10.1128/JB.00195-21.
4. Baba T, Ara T, Hasegawa M, Takai Y, Okumura Y, et al. (2006) Construction of *Escherichia coli* K-12 in-frame, single-gene knockout mutants: the Keio collection. Mol Syst Biol 2: 2006.0008.
5. Brochu J, Vlachos-Breton É, Sutherland S, Martel M, Drolet M. (2018) Topoisomerases I and III inhibit R-loop formation to prevent unregulated replication in the chromosomal Ter region of *Escherichia coli*. PLoS Genet. Sep 17;14(9):e1007668. doi: 10.1371/journal.pgen.1007668.
6. Drolet M, Phoenix P, Menzel R, Massé E, Liu LF et al. (1995) Overexpression of RNase H partially complements the growth defect of an *Escherichia* *coli* delta *topA* mutant: R-loop formation is a major problem in the absence of DNA topoisomerase I. Proc Natl Acad Sci U S A . 92:3526-3530.
7. Stupina VA, Wang JC. (2005) Viability of *Escherichia coli* *topA* mutants lacking DNA topoisomerase I. J Biol Chem. 280:355-360.
8. Usongo V, Drolet M. (2014) Roles of type 1A topoisomerases in genome maintenance in *Escherichia coli*. PLoS Genet. Aug 7;10(8):e1004543. doi: 10.1371/journal.pgen.1004543.
9. Massé E, Drolet M. (1999) *Escherichia coli* DNA topoisomerase I inhibits R-loop formation by relaxing transcription-induced negative supercoiling. J Biol Chem. 274:16659-16664.
10. Lavasani LS, Hiasa H. (2001) A ParE-ParC fusion protein is a functional topoisomerase. Biochemistry. Jul 24;40(29):8438-43.
11. Zumstein L, Wang JC. (1986) Probing the structural domains and function *in vivo* of *Escherichia coli* DNA topoisomerase I by mutagenesis. J Mol Biol. Oct 5;191(3):333-40.
12. Ahumada A, Tse-Dinh YC. (2002) The role of the Zn(II) binding domain in the mechanism of *E. coli* DNA topoisomerase I. BMC Biochem. May 29;3:13. doi:10.1186/1471-2091-3-13.
13. Cao N, Tan K, Zuo X, Annamalai T, Tse-Dinh YC. (2020) Mechanistic insights from structure of *Mycobacterium smegmatis* topoisomerase I with ssDNA bound to both N-and C-terminal domains. Nucleic Acids Res. May 7;48(8):4448-4462. doi:10.1093/nar/gkaa201.
